# Mathematical models of neuronal growth

**DOI:** 10.1007/s10237-021-01539-0

**Published:** 2022-01-07

**Authors:** Hadrien Oliveri, Alain Goriely

**Affiliations:** Mathematical Institute, University of Oxford, Oxford, OX2 6GG UK

**Keywords:** Neurodevelopment, Axon, Neurite, Growth, Biomechanics, Mathematical modeling, 92C05, 92C10, 92C20, 92C15, 92C37, 92C17

## Abstract

The establishment of a functioning neuronal network is a crucial step in neural development. During this process, neurons extend neurites—axons and dendrites—to meet other neurons and interconnect. Therefore, these neurites need to migrate, grow, branch and find the correct path to their target by processing sensory cues from their environment. These processes rely on many coupled biophysical effects including elasticity, viscosity, growth, active forces, chemical signaling, adhesion and cellular transport. Mathematical models offer a direct way to test hypotheses and understand the underlying mechanisms responsible for neuron development. Here, we critically review the main models of neurite growth and morphogenesis from a mathematical viewpoint. We present different models for growth, guidance and morphogenesis, with a particular emphasis on mechanics and mechanisms, and on simple mathematical models that can be partially treated analytically.

## Introduction

**Fig. 1:**
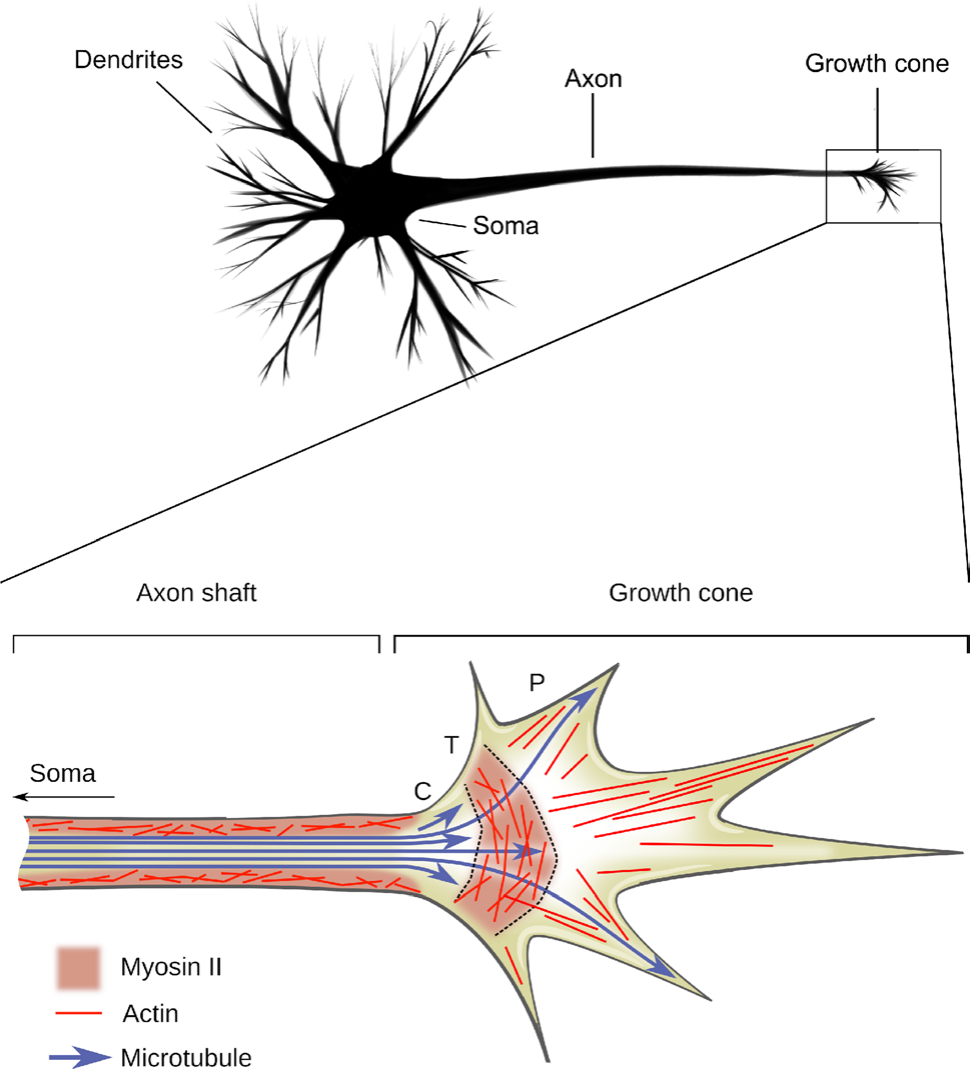
Schematic of a typical neuron showing the soma, the dendrites and the axon with its growth cone, prior to synaptogenesis. Close-up shows a schematic representation of the cytoskeleton in a migrating neurite. The shaft (on the left) is essentially composed of cross-linked microtubules surrounded by an actomyosin sheath. The growth cone is a lamellipodium-like structure that confers motility to the neurite, probes its environment using sensory filopodial protrusions and produces traction forces applied to the shaft promoting neurite extension

The human brain contains an estimated $\sim$86 billion neurons (Azevedo et al. 2009) that interconnect through numerous slender processes, *axons* and *dendrites*, globally known as *neurites*. A typical nerve cell has one long axon, that transmits nervous signals, and multiple branching dendrites, that receive signals (Striedter 2016, p. 35), see Fig. 1. During early neuron development, multiple neurites sprout from the cell body (or *soma*). During this phase, typically, one incipient neurite comes to outgrow the others and differentiates into an axon (Kiryushko et al. 2004). This axon then migrates through the extracellular matrix in order to make synaptic connections with other distant neurons or tissues.

It is now understood that this fundamental and robust process depends on many different genetic, biochemical and physical factors. Mathematical modeling has become a prominent actor of developmental biology and is crucial to achieve a rational organization of the intricate physical processes involved in neuronal development. Here, we provide a critical, but not exhaustive, review of about three decades of mathematical modeling of axon and dendrite development. This paper is meant to complement existing reviews that emphasize physical and chemical mechanisms of growth and guidance (O’Donnell et al. 2009; Franze and Guck 2010; Franze 2020; Seo et al. 2020; Suter and Miller 2011; Miller and Suter 2018; Mortimer et al. 2008; McCormick and Gupton 2020; Abuwarda and Pathak 2020; Dickson 2002; Franze et al. 2013; Dent and Gertler 2003). computational and numerical models (Simpson et al. 2009; Kiddie et al. 2005; Graham and Van Ooyen 2006; Van Ooyen 2011, 2003; Ascoli 2002; Maskery and Shinbrot 2005; Goodhill 2018), or mechanical models (Goriely et al. 2015a). Our particular emphasis is on analytically tractable mathematical models that provide key insights into possible mechanisms and properties emerging from nonlinear couplings, and the combination of different physical phenomena such as material transport, growth and mechanics.

## Biological background

Neurite motility is mediated by the *growth cone* (Fig. 1), a highly dynamic actin-supported extension at the tip of the neurite that performs both sensory and locomotory functions. Within the growth cone, the cytoskeleton is mostly constituted of actin filaments (F-actin) that organize in a lamellipodium-like structure. Centrifugally arranged actin filaments polymerize at the periphery of the growth cone (P-domain) and undergo a treadmill-like retrograde flow mediated by myosin II motors in a transition zone (T-domain), where actin depolymerizes. Combined with focal adhesions that mechanically couple F-actin with the substrate (Chan and Odde 2008), this continuous movement generates forces that propagate through a connecting region (C-domain) to the trailing part of the neurite, the *neurite shaft* (Franze 2020), and mediate neurite motility.

The neurite shaft is a filament-like section that contains the neurite cytoplasmic compartment (Kevenaar and Hoogenraad 2015), composed mainly of stabilized microtubules cross-linked by microtubule-associated proteins (MAP) (Maccioni and Cambiazo 1995), F-actin and neurofilaments. Microtubules are vital for intracellular trafficking (Caviston and Holzbaur 2006) and for the neurite’s structural integrity. The microtubule array is itself coated with an actomyosin cortical sheath generating contractile stresses (Franze 2020; Jülicher et al. 2007) that under certain circumstances may cause neurite retraction (Recho et al. 2016; Franze et al. 2009a). The inner and outer layers of the neurite shaft are connected through special proteins ensuring force transmission between the two structures (Coles and Bradke 2015).

We define neurite growth as the irreversible elongation of the neurite shaft supported by addition of new cellular material (Goriely 2017; Goriely et al. 2015a). The precise mechanisms underlying neurite growth have been debated for about 40 years and remain controversial. An important idea in the 80s–90s was that microtubules are stationary during neurite extension (Miller and Joshi 1996; Lim et al. 1990; Okabe and Hirokawa 1990; Takeda et al. 1995; Sabry et al. 1995) and assemble at the neurite distal tip to elongate the shaft (Dent and Gertler 2003; Goldberg and Burmeister 1986; Bamburg et al. 1986), a process regulated through growth cone traction forces (Buxbaum and Heidemann 1988, 1992; Lamoureux et al. 1989; Heidemann et al. 1990). Thus, a vast literature has focused on *slow axonal transport* (Miller and Heidemann 2008; Roy 2014, 2020), the general process by which cytoskeletal proteins, such as neurofilaments, actin and tubulin (the building unit of microtubules), transit along the neurite. The mechanisms of slow axonal transport have been debated for decades and remain relatively poorly understood (for a review of models, see Bressloff and Newby 2013).

To date, direct observation of this tip growth process is lacking. Moreover, this hypothesis seems in apparent contradiction with the finding that neurites isolated from a substrate may stretch rapidly when subject to externally applied deformation or force, a process which does not seemingly involve the growth cone (Dennerll et al. 1989; Bray 1984; Rajagopalan 2010; Zheng et al. 1991; Lamoureux et al. 2010; Heidemann et al. 1997).

The idea that the shaft is stationary has been dramatically challenged by more recent investigations which have clearly evidenced that the neurite shaft stretches during growth cone migration (Miller and Suter 2018; Athamneh et al. 2017; Lamoureux et al. 2010; O’Toole et al. 2008a; Miller and Sheetz 2006). These observations are partly consistent with a viscoelastic creep of the shaft under prolonged traction, mediated by adhesion (O’Toole et al. 2008a). It is now generally understood that neurite growth is largely controlled by mechanics and depends on the complex interplay of active pushing and pulling forces mediated by growth cone traction, microtubule assembly, shaft viscoelasticity and contractility, and adhesion (Recho et al. 2016; Franze 2020; Miller and Suter 2018; Suter and Miller 2011).

To reach their functional targets, axons must extend along precise paths. This early phase, known as *axon guidance* (or *pathfinding*), crucially relies on cues from the environment which are processed to instruct growth cone trajectory. This includes *chemotaxis* primarily, i.e., guidance of axons by gradients of diffusing chemicals like Slits or Netrins (Mortimer et al. 2008; Plachez and Richards 2005; Chilton 2006); but also *haptotaxis*, i.e., guidance by gradients in adhesion or substrate-bound chemicals (Sundararaghavan et al. 2011); *durotaxis* (or *mechanotaxis*), i.e., guidance by gradients in substrate stiffness (Abuwarda and Pathak 2020; Koser et al. 2016; Espina et al. 2021); *electrotaxis* (or *galvanotaxis*), i.e., guidance by electric field (Yao and Li 2016; Hamid and Hayek 2008; Gokoffski et al. 2019; Shapiro et al. 2005); *curvotaxis*, i.e., guidance by substrate curvature (Smeal et al. 2005); or guidance assisted by guidepost cells such as radial glial cells (Franze et al. 2009b; Rakic 1972) or Schwann cells (Thompson and Buettner 2006). The growth cone is the main sensory structure at play in all these guidance modalities.

Once an axon has reached its target, typically neurons or other target effector cells, it forms synapses (*synaptogenesis*) and is therefore bound to these cells. Then, the axon shaft must extend to accommodate the subsequent growth of the surrounding tissue. In this *stretch growth* phase, spectacularly fast axon extension may take place, dictated by the growth of the animal’s body (Smith 2009) and governed by forces (Pfister et al. 2004). It is now understood that maintaining a proper tension along the neurite is important in normal function (Siechen et al. 2009). Body growth imposes strong kinematic constraints on the neuron, which must then sustain sufficient cell material supply while performing fast remodeling along the stretched neurite in order to maintain its structural integrity and avoid traumatic levels of strain. Several authors have modeled and studied this problem, e.g., O’Toole and Miller (2011); O’Toole et al. (2008b); Purohit and Smith (2016), who have examined the role of stretching in controlling the flux of material along the shaft (see Sect. 3.3). However, this problem remains poorly understood; yet, it is nonetheless crucial for instance in the design of nerve regeneration therapies.

## Modeling neuritic growth

Neurite growth involves different coupled mechanisms that have not been fully resolved. Initially, growth was approached from the point of view of transport, mass supply and addition, i.e., as a process entirely governed by the availability of given substances required for growth. In particular, many authors have focused extensively on the transport and assembly of tubulin at the tip of the neurite, long viewed as the main growth determinant. Alternatively, experiments have clearly shown that neurons grow in response to applied mechanical forces. It is then natural to model growth in terms of forces, strains, stresses and rheology. Here, we consider both approaches, starting with transport models.

### Transport-limited growth

By definition, growth is related to the notion of mass uptake (Goriely 2017). One of the main growth determinants is soluble tubulin that was assumed to assemble at the end of the neurite. In young neurites, the new cell material is supplied by the soma. Therefore, the cell must first produce new tubulin units (and other cell constituents) and then carry them along the shaft, using diffusion and active transport, to reach the end of the neurite where growth takes place. This process is at the core of numerous mathematical works (see the reviews by Kiddie et al. 2005; Graham and Van Ooyen 2006; Van Ooyen 2011, 2003).

#### Zero-dimensional models

**Fig. 2:**
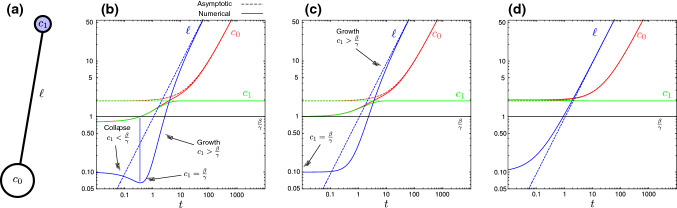
Two-compartment model for neurite growth. **a** Cartoon of a neuron represented by two compartments, representing the soma and the growth cone (associated with respective concentrations $c_0$ and $c_1$). **b**–**d** Log–log plot of $\ell$, $c_0$ and $c_1$ versus time *t* based on (6–8), for different initial concentrations, from left to right: **b** $c_0(0)=c_1(0)=0.8<\beta /\gamma$; **c** $c_0(0)=c_1(0)=1=\beta /\gamma$; **d** $c_0(0)=c_1(0)=2>\beta /\gamma$. Other parameter values: $\ell _0=0.1$, $\alpha =10$, $\beta =1$ and $\gamma =1$

A first approach consists in modeling the neurite by a small number of compartments (Janulevicius et al. 2006; Samuels et al. 1996; Purohit and Smith 2016; Van Veen and Van Pelt 1994; Lin et al. 2020). Each compartment is associated with one or more chemical concentrations and exchanges material with other compartments via diffusion and/or active transport by motor proteins. For instance, in a model such as the one proposed by Van Veen and Van Pelt (1994), the neurite is modeled by two compartments, the soma, where the tubulin dimer concentration $c_\text {0}$ is defined, and the growth cone with tubulin concentration $c_\text {1}$ (Fig. 2a). The two compartments are *virtually* separated by a distance $\ell$ that represents the neurite length.

Growth relies on assembly and disassembly of tubulin at the neurite tip, following a polymerization reaction of the type

1 $$\begin{aligned} T_n + T_1 {\mathop {\rightleftharpoons }\limits ^{k^+}_{k^-}} T_{n+1}, \end{aligned}$$

where $T_n$ denotes a microtubule of length *n* for $n >1$, or a tubulin dimer if $n=1$; and $k^+$ and $k^-$ are rate constants. This process implies the kinetic law

2 $$\begin{aligned} \frac{1}{e}\frac{\text {d}{\ell }}{\text {d}{t}} = k^+ c_1 - k^-, \end{aligned}$$

where *e* represents the typical elongation due to the addition of one tubulin dimer (in unit length per mole of tubulin). Growth is coupled to $c_1$ since it consumes tubulin ($k^+$), and conversely, disassembled tubulin is reintroduced ($k^-$) in the available pool of tubulin at the tip. In addition, tubulin is supplied by the soma through diffusion. A Fickian flux *J* is assumed, where tubulin diffuses with diffusion constant *D* as

3 $$\begin{aligned} J = AD\frac{c_1 - c_0}{\ell }, \end{aligned}$$

with *A* the cross-sectional area of the neurite’s cytoplasmic compartment. Summing up all contributions, we obtain

4 $$\begin{aligned} V\frac{\text {d}{c_\text {1}}}{\text {d}{t}} =J + k^- - {k^+} c_1, \end{aligned}$$

with *V* a typical volume where reaction takes place. To close the system, we take into account the production of tubulin by the soma with rate *S*:

5 $$\begin{aligned} V\frac{\text {d}{c_\text {0}}}{\text {d}{t}} = S - J , \end{aligned}$$

where we have used the same volume *V* introduced previously. Choosing the time, length and concentration units to be, respectively, *A*/*D*, *SAe*/*D* and *SA*/*VD*, we obtain the non-dimensionalization

6 $$\begin{aligned} \frac{\text {d}{{\tilde{c}}_0}}{\text {d}{{\tilde{t}}}}&= 1 - \alpha \frac{{\tilde{c}}_1 - {\tilde{c}}_0}{\tilde{\ell }}, \end{aligned}$$

7 $$\begin{aligned} \frac{\text {d}{{\tilde{c}}_1}}{\text {d}{{\tilde{t}}}}&= - \gamma {\tilde{c}}_1 + \beta + \alpha \frac{{\tilde{c}}_1 - {\tilde{c}}_0}{\tilde{\ell }}, \end{aligned}$$

8 $$\begin{aligned} \frac{\text {d}{\tilde{\ell }}}{\text {d}{{\tilde{t}}}}&= \gamma {\tilde{c}}_1 - \beta , \end{aligned}$$

where $\alpha ={AD}/{Se}$, $\beta ={k^-}/{S}$ and $\gamma =Ak^+/VD$ are dimensionless. For simplicity, we henceforth drop the tildes and work with dimensionless variables.

We stress that the length of the neurite $\ell$ is a dynamical variable, and not a spatial dimension of the problem. Hence, we have an autonomous system of three nonlinear ordinary differential equations for the variables $c_0$, $c_1$ and $\ell$, which can be analyzed using dynamical systems theory. This system has an exact solution given by

9 $$\begin{aligned} c_0\left( t \right) =c_{00}+c_{01}t,\quad c_1\left( t \right) =c_{00},\quad \ell \left( t \right) =v t, \end{aligned}$$

with

10 $$\begin{aligned}&c_{00}=\frac{\beta }{\gamma }+\frac{1}{2\gamma }\left( \sqrt{\alpha (\alpha +4)}-\alpha \right) , \end{aligned}$$

11 $$\begin{aligned}&c_{01}=1-\frac{1}{2}\left( \sqrt{\alpha (\alpha +4)}-\alpha \right) , \end{aligned}$$

12 $$\begin{aligned}&v=\frac{1}{2} \left( \sqrt{\alpha (\alpha +4)}-\alpha \right) , \end{aligned}$$

see dashed lines in Fig. 2b–d. This solution also fully captures the asymptotic dynamics, and for positive initial conditions we have that as $t\rightarrow \infty$, $c_0\sim c_{01}t$, $\ell \sim vt$ and $c_1\rightarrow c_{00}$. Another interesting feature of the model that can be observed from (8) is that for small initial concentrations $c_1(0)< \beta /\gamma$ and $c_0(0)>c_1(0)$, i.e., when disassembly dominates over assembly, we have ${\dot{\ell }}<0$, and hence, we have a transient neurite collapse. However, under the same conditions, we have $\dot{c}_1>0$. Hence, $c_1$ will increase in time until ${\dot{\ell }}\ge 0$ at which time the growth of the neurite resumes.

A drawback of the solution (9) is that both soma concentration $c_0$ and length $\ell$ increase without bound. In reality, tubulin dimers may inhibit the translation of new tubulin (Gay et al. 1989), and thus, cells have a limit on $c_0$ and may generally use a feedback loop to control it. It is also clear that the transported proteins have finite half-lives, which imposes a theoretical limit on neurite length (Miller and Samuels 1997; McLean et al. 2004).

**Fig. 3:**
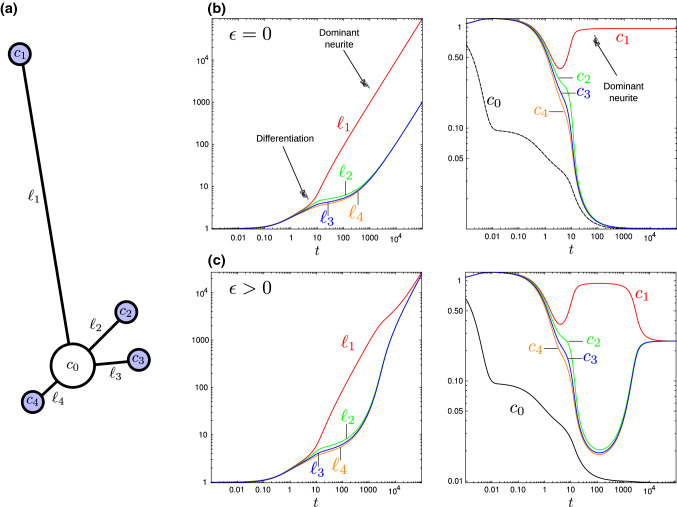
Multiple neurites. **a** Compartmental model of a neuron composed of one soma (white) and $N=4$ neurites with tip concentrations $c_i$ (blue). **b**–**c** Example simulations, based on Samuels et al. (1996), with $\epsilon =0$ **b** and $\epsilon =0.1$ **c**. Log–log plots show neurite lengths versus time (left) and concentrations versus time (right). Parameter values for both simulations: $\alpha =10$, $\beta =0$, $\gamma =1$, $\delta =100$; initial concentrations: $c_i(0)=1$ for all $i=0,1,2,3,4$; initial lengths: $\ell _1=1$, $\ell _2=0.99$, $\ell _3=0.98$ and $\ell _4=0.97$

An interesting extension of the two-compartment model is the application to multiple neurites (Van Ooyen et al. 2001; Samuels et al. 1996; Toriyama et al. 2010; Fivaz et al. 2008) or neurites with multiple branches (Li et al. 1992; Van Veen and Van Pelt 1994; Hjorth et al. 2014). For the sake of illustration, consider $N>1$ neurites growing from the same soma, as illustrated in Fig. 2a. Defining $c_i$ to be the concentration at the tip of the *i*th neurite of length $\ell _i$, the flux for neurite *i* is defined following Samuels et al. (1996) as

13 $$\begin{aligned} J_i = \alpha \frac{c_i - c_0}{\ell _i} + \epsilon c_0 + \zeta \frac{\text {d}{\ell _i}}{\text {d}{t}} c_0, \end{aligned}$$

where we have added a growth-independent transport controlled by a parameter $\epsilon$, and a growth-dependent transport controlled by a parameter $\zeta$. The last term models a growth-sensitive feedback loop in which a faster-growing neurite receives a larger influx of tubulin. Hence, the *N*-compartment model leads to a system of $2N+1$ differential equations for $c_0$, $c_i$ and $\ell _i$. The last term in (13) introduces nonlinearity and possible instabilities. Indeed, this new augmented system is very sensitive to the choice of initial lengths (but relatively insensitive to differences in initial concentrations). Since neurites arise from the soma at different times, we initiate the simulation with different initial lengths $\ell _i(0)$. For large values of the feedback parameter $\zeta$ (here $\zeta =100$) and neglecting growth-independent transport ($\epsilon =0$), a winner-takes-all instability is observed, where one neurite comes to outgrow the others by monopolizing tubulin resources. An example of such phenomenon is shown in Fig. 3b where we have simulated $N=4$ neurites with initial lengths $\ell _0=1$, 0.99, 0.98 and 0.97. This mechanism provides insight into the mechanism by which a neurite differentiates into an axon during early neuronal development. Note, however, that as soon as $\epsilon >0$, the shortest neurites, whose growth is initially inhibited by the dominant neurite, catch up at later times as concentrations become homogeneous across the *N* tips, as shown in Fig. 3c with $\epsilon =0.1$.

The prediction that the initially longest neurite almost systematically becomes the axon is probably inaccurate in reality, as exemplified by situations where an initially shorter neurite outgrows longer ones (see, for instance, Fig. 6 in Fanti et al. 2008). In fact, the initial length of neurites is one among many other endogenous and exogenous factors that may participate in breaking the symmetry. Nevertheless, this simple model, based on the classic concept of patterning by autocatalysis and lateral inhibition (Turing 1952; Gierer and Meinhardt 1972; Meinhardt and Gierer 2000), shows how a simple competition mechanism can trigger symmetry breaking and prevent the formation of multiple axons (an idea further supported by later investigations, e.g., Fivaz et al. 2008; Toriyama et al. 2010; Takano et al. 2015, 2017; Inagaki et al. 2011).

#### One-dimensional models

**Fig. 4:**
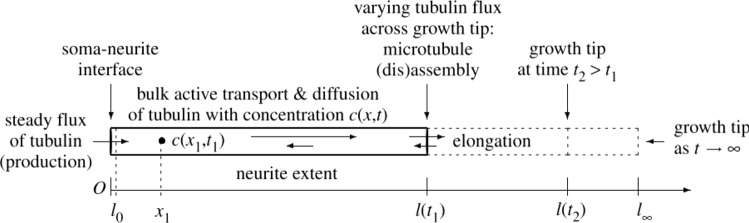
Schematic showing the geometry of the partial differential equation problem and the principal mechanisms involved. Image reproduced from McLean et al. (2004) (with permission from *The Royal Society*)

The main postulate of compartmental approaches is that the concentration profile along a neurite is linear and defined by only two quantities $c_i$ and $c_0$ at both ends of the neurite. For a long neurite such as an axon, diffusion and transport are not instantaneous, and proteins may degrade significantly before reaching the tip. Hence, delay and spatial heterogeneity in cellular material concentrations will appear along the neurite. Thus, the coarse two-compartment approximation (3) needs to be refined by considering the value of the concentration at every point along the neurite. A possible generalization of the compartmental approach consists in considering neurites made up of many concatenated compartments representing short contiguous portions of the neurite shaft, where each segment exchanges solutes with its adjacent neighbors (Hely et al. 2001; Kiddie et al. 2005; Graham and Van Ooyen 2001, 2004; Hjorth et al. 2014). Bulk growth can be simulated by growing and subdividing each compartment. Alternatively, new compartments may be added at the tip, to simulate tip growth. Mathematically, it is interesting to consider the continuum limit of these approaches through a proper partial differential equations formulation of the fundamental convection–diffusion problem, and then to use numerical discretization methods when needed (Smith and Simmons 2001; McLean et al. 2004; McLean and Graham 2004, 2006; Graham et al. 2006; Diehl et al. 2014, 2016; Zadeh and Shah 2010). Figure 4, reproduced from McLean et al. (2004), gives the geometry of the problem and the principal mechanisms involved. The continuity equations express conservation of each substance considered (e.g., proteins, vesicles, mitochondria). Here, we consider one diffusing substance (e.g., tubulin) with concentration *c* obeying

14 $$\begin{aligned} \frac{\partial {c}}{\partial {t}} + \frac{\partial {J}}{\partial {x}} = S\left( x,t \right) . \end{aligned}$$

In this expression *J* is the anterograde flux of material (from the soma and oriented toward increasing *x*), and *S* represents any source or sink. The flux $J=J_\text {d}+J_\text {a}$ includes the regular diffusion $J_\text {d} =D {\partial {c}}/{\partial {x}}$ with *D* the molecular diffusivity, and a kinesin-mediated flow $J_\text {a}=ac$ with *a* an effective active transport velocity.

Typically, transported proteins may also decay with rate constant $\kappa$, which is included into a sink term $S=-\kappa c$. In the absence of diffusion ($D=0$), (14) supports a steady state solution $c=c_0 \exp (-\kappa x/a)$, where $c_0$ is the concentration of tubulin in the soma. This solution identifies a characteristic length of $a/\kappa$ that may be interpreted as a maximal length for the neurite (Miller and Samuels 1997).

Several boundary constraints have been used. For the proximal boundary conditions, we can specify at the soma, either the concentration ${\left. c \right| _{0,t}}={\tilde{c}}_0\left( t \right)$ (McLean and Graham 2004; Diehl et al. 2014, 2016) or the flux ${\left. {\partial {c}}/{\partial {x}} \right| _{0,t}}=\epsilon _0 c\left( 0,t \right)$ (McLean et al. 2004; McLean and Graham 2006; Graham et al. 2006). The distal boundary condition is given by a flux that models the absorption of tubulin by the growth process. McLean et al. (2004) use, for instance, a flux boundary constraint of the form

15 $$\begin{aligned} \left. {\partial {c}}/{\partial {x}}\right| _{x=\ell \left( t \right) ,t}=-\epsilon _\ell c\left( \ell \left( t \right) ,t \right) + \zeta _\ell , \end{aligned}$$

where $\epsilon _\ell$ and $\zeta _\ell$ are constants related to assembly and disassembly of tubulin, respectively. Growth is then modeled by adapting (2) in terms of the Stefan condition,

16 $$\begin{aligned} \frac{1}{e}\frac{\text {d}{\ell }}{\text {d}{t}} = k^+ c\left( \ell \left( t \right) ,t \right) - k^-, \end{aligned}$$

which expresses the displacement of the distal boundary of the domain, depending on the availability of tubulin at the neurite tip. Moving-boundary problems are notoriously difficult to solve. However, in one dimension, the domain size can be fixed through the change of variable $y \left( x,t \right) = x / \ell \left( t \right) \in \left[ 0,1 \right]$. Equation (14) is then recast as

17 $$\begin{aligned} \frac{\partial {{\tilde{c}}}}{\partial {t}} + \frac{1}{\ell }\left( a- y\frac{\text {d}{\ell }}{\text {d}{t}} \right) \frac{\partial {{\tilde{c}}}}{\partial {y}} -\frac{D}{\ell ^2}\frac{\partial ^2 {{\tilde{c}}}}{\partial {y}^2} = S\left( y \ell , t \right) , \end{aligned}$$

with ${\tilde{c}}\left( y,t \right) {:}{=}c\left( \ell y,t \right)$ for all $y\in \left[ 0,1 \right]$, and where $\ell \left( t \right)$ is governed by (16).

**Fig. 5:**
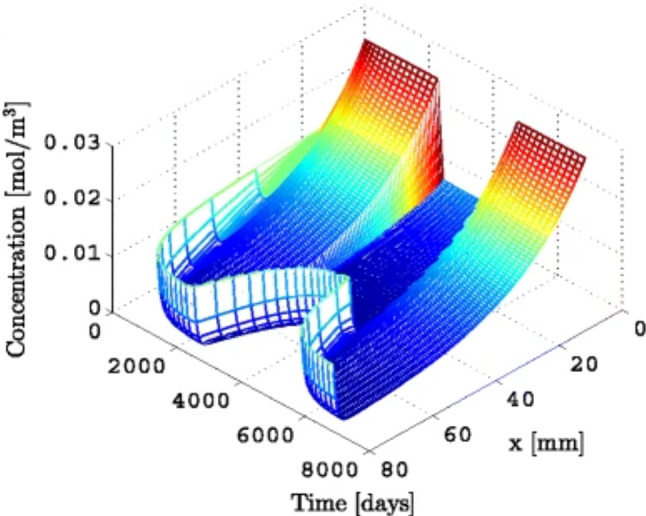
Axon growth and collapse as a result of a piecewise constant soma concentration. The tubulin concentration $c\left( x, t \right)$ in the axon is plotted over time and space. Image reproduced from Diehl et al. (2016) (with permission from *Springer*)

The full time-dependent problem is analytically intractable in general, and numerical approaches are necessary (see, for instance, the simulation by Diehl et al. 2016, shown in Fig. 5). However, focusing on the steady regime, McLean et al. (2004); McLean and Graham (2004); Graham et al. (2006) show that the neurite reaches a uniquely defined final length when tubulin degradation is considered (see also Miller and Samuels 1997) and grows without bound otherwise (as in Van Veen and Van Pelt 1994).

Overall, these 0D and 1D models generally predict a decrease in tubulin concentration toward the tip, as in the 0D Van Veen–Van Pelt model or the 1D McLean–Graham approach. It can be noticed, however, that the boundary condition (15) used by McLean and Graham (2004) actually neglects the moving boundary and is not consistent with the expression of the flux. To resolve this issue, Diehl et al. (2014) represent the growth cone as a finite volume with tubulin concentration $c_\text {gc}$, where tubulin polymerization is modeled separately through an ordinary differential equation. Therefore, the distal boundary constraint consists in enforcing the continuity of *c* at the interface, i.e., $c\left( \ell \left( t \right) ,t \right) =c_\text {gc}$ (rather than a flux). This modification of the original model results in very different, non-monotonic, concentration profiles that show an accumulation of tubulin near the tip when transport is faster than elongation (Fig. 5). Experimentally, this is reminiscent of the distal accumulation of MAPs reported by Black et al. (1994), but is, however, inconsistent with the nearly uniform concentration of mitochondria observed by O’Toole et al. (2008b) (see Sect. 3.3) that may be explained by a slowdown of transport toward the tip (as also suggested by Hoffman et al. 1985; Watson et al. 1989).

The general approach presented here is based on the idea that elongation is governed by microtubule assembly at the tip, and that high tubulin concentration causes faster growth (16). This assumption has not been directly demonstrated experimentally and is partly challenged by recent observations, such as those made by Ren and Suter (2016) in pausing *Aplysia* neurites (see also the comment by Miller and Suter 2018). When the growth cone pauses, it rapidly increases in cell content due to transport, which correlates with a rapid increase in growth cone size. Yet, the growth cone neck does not advance, indicating that neurite growth is limited by growth cone advancement, rather than transport and microtubule assembly. In addition, this view neglects other rate-limiting constituents such as MAPs, neurofilaments, mitochondria or membrane lipids, as well as mechanical forces. Several models have addressed the modulation of tubulin assembly by forces, such as Buxbaum and Heidemann (1988, 1992), or, more recently, Purohit (2015), who modified the Van Veen–Van Pelt model to include mechanical regulation of tip growth. In the next section, the focus is on the mechanical models of neurite growth.

### Mechanically mediated growth

An important aspect of neuronal development involves regulation through physical forces (Mutalik and Ghose 2020; Franze 2020; Suter and Miller 2011; Franze et al. 2013; Miller and Suter 2018). In particular, the initiation and growth of neurites may be induced directly by forces applied on the neurite (Heidemann and Buxbaum 1990; Bray 1984; Zheng et al. 1991; Dennerll et al. 1989). For example, Zheng et al. (1991) showed that the initiation of axons of chick sensory neurons can be triggered experimentally by the application of tension on the surface of the cell body. Then, further elongation can be induced by towing the neuron with a glass needle. The deflection of the needle tip can be used as a control to apply constant force. Strikingly, these experiments demonstrate that growth rates increase with applied forces. While it remains unclear whether neurites undergo tip growth, an alternative paradigm has emerged more recently, where forces drive the fluid-like expansion of the neurite shaft, while transport provides the material necessary to support elongation through mass addition (Miller and Suter 2018; Suter and Miller 2011).

To model the role of forces acting on the neuron and its internal components, we start with a system composed of a single isolated unit (zero-dimensional models) subject to forces, before considering more complex models with multiple components and with spatial variations in one dimension. In contrast to Sect. 3.1, the problem of material supply will be considered here as non-limiting.

#### Zero-dimensional models

It is well appreciated that isolated neurites respond elastically under rapid plucking (Heidemann et al. 1997), and creep irreversibly without thinning when subject to prolonged overstretch (Dennerll et al. 1989; Bray 1984; Heidemann et al. 1997; Pfister et al. 2004). Typical experimental studies of mechanically mediated neurite growth consider isolated neurites subject to imposed displacement or force. To model the mechanical response of neurites in such condition, *spring-and-dashpot*-type models have been used (Dennerll et al. 1989; Bernal et al. 2007; Li and Qin 1996; O’Toole et al. 2015; Lin et al. 2020) where the entire neurite is represented as a single unit with no spatial variation. A more detailed approach emphasized here consists in viewing the neurite as a *morphoelastic* tubular compartment (Anthonisen and Grütter 2019; Goriely et al. 2015a; Goriely 2017; Wang and Kuhl 2019; Holland et al. 2015). Morphoelasticity (Goriely 2017) is an extension of nonlinear elasticity which provides a general mechanical description of growth based on a multiplicative decomposition of the deformation gradient (Rodriguez et al. 1994). It is a natural framework to represent nonlinear deformations along the neurite, as well as more complex multidimensional processes such as axon beading (Riccobelli 2021) or turning (García-Grajales et al. 2017). In particular, morphoelasticity naturally takes into account changes in cross-sectional area, which has been used experimentally as an indicator of growth (O’Toole et al. 2008a; Lamoureux et al. 2010), as well as arbitrary nonlinear elastic response functions. The application of morphoelasticity to axon growth, elasticity and damage is extensively discussed in Goriely et al. (2015a). In particular, one core idea of morphoelastic modeling that differs in spirit with modeling through spring and dashpots is the particular focus on biological mechanisms. The extra variables introduced in the theory represent effects such as remodeling and addition of mass. The overall behavior of such systems under loads, namely its rheology, is then an emergent property rather than a choice of constitutive law based on experimental observations.

**Fig. 6:**
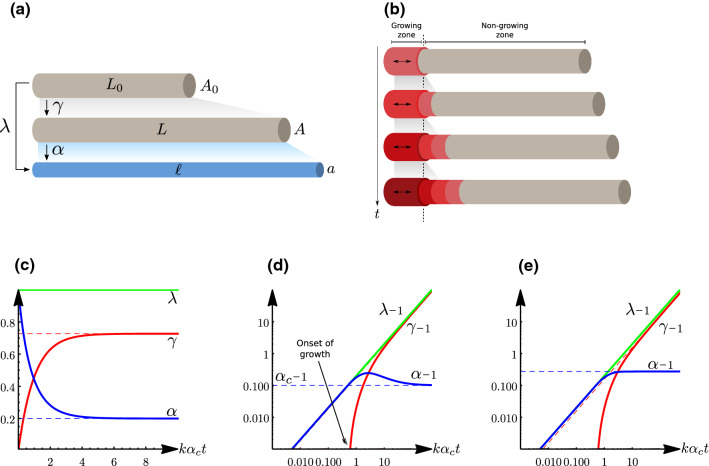
Zero-dimensional morphoelastic model of neurite growth **a** Multiplicative decomposition of the stretch $\lambda$ from initial (stress-free) configuration (with length $L_0$), to intermediate, stress-free configuration (with length *L*), to current configuration (with length $\ell$). The multiplier $\gamma$ accounts for the anelastic deformation of the neurite, while $\alpha$ quantifies the elastic stretch that results in mechanical stress. **b** Cartoon illustrating the localized growth model where growth takes place only in the axon hillock. The basal zone (red) is the only part that grows. Growth is frozen when the considered material point exists the hillock. **c** *Stretch-and-hold* experiment $\lambda \left( t \right) =\lambda _0$. Plot of $\lambda \left( t \right)$, $\alpha \left( t \right)$ and $\gamma \left( t \right)$ versus normalized time $k\alpha _\text {c}t$. All stretches are normalized as $x\rightarrow \left( x-1 \right) /\left( \lambda _0-1 \right)$. **d**, **e** *Speed-controlled* traction experiment $\lambda \left( t \right) =1+t/\tau '$ simulated with both **d** the exponential growth model (19) and **e** the linear growth model (30). Log–log plot of $\lambda \left( t \right) -1$, $\alpha \left( t \right) -1$ and $\gamma \left( t \right) -1$ vs. normalized time $k\alpha _\text {c}t$. Dashed lines show the asymptotic approximations. Parameters: $\alpha _\text {c}=1.1$, $\lambda _0=1.5$, $k=1$, $\tau '=5 \tau$

A neurite is represented initially by a homogeneous stress-free cylinder $\varOmega _0$ of cross-sectional area $A_0$ and length $L_0$ (Fig. 6a). We assume that the neurite is fixed at one end (defined as the soma). At a later time *t*, the current (observed) configuration of the neurite is another cylinder $\varOmega$ with cross section $a\left( t \right)$ and length $\ell \left( t \right)$. We denote by $\mathbf {X}_0=\left( X_0,Y_0,Z_0 \right) \in \varOmega _0$ and $\mathbf {x}=\left( x,y,z \right) \in \varOmega$ the positional vectors in the initial and current configuration, respectively. Since we do not allow spatial variations, the deformation gradient $\mathbf {F} {:}{=}{\partial {\mathbf {x}}}/{\partial {\mathbf {X}_0}}$ is uniform across the whole domain and is given by the tensor $\mathbf {F} = {{\,\mathrm{diag}\,}}\left( \lambda , \lambda ^\perp , \lambda ^\perp \right)$, with $\lambda$ and $\lambda ^\perp$ the longitudinal and transverse stretches.

The main postulate of morphoelasticity consists in expressing the deformation gradient in terms of two tensors: a growth tensors $\mathbf {G}$ due to the change of volume or internal reconfiguration, which is stress-free, and an elastic tensor $\mathbf {A}$, that characterizes the elastic response, and generates stress (Rodriguez et al. 1994). Constitutively, these two tensors are multiplicatively coupled via

18 $$\begin{aligned} \mathbf {F}=\mathbf {A}\cdot \mathbf {G}, \end{aligned}$$

where $\mathbf {A}={{\,\mathrm{diag}\,}}\left( \alpha ,\alpha ^\perp ,\alpha ^\perp \right)$ and $\mathbf {G}={{\,\mathrm{diag}\,}}\left( \gamma ,\gamma ^\perp ,\gamma ^\perp \right)$. The growth deformation transforms the initial cylinder $\varOmega _0$ into a new intermediate cylinder $\varOmega _\text {g}\left( t \right)$ of length $L\left( t \right) =\gamma \left( t \right) L_0$ and cross-sectional area $A\left( t \right) =\gamma ^\perp \left( t \right) A_0$, whereas the elastic part of the deformation transforms the grown cylinder $\varOmega _\text {g}\left( t \right)$ into the observed cylinder $\varOmega \left( t \right)$ with $\ell =\alpha ^\perp \left( t \right) L\left( t \right)$ and $a\left( t \right) =\alpha ^\perp \left( t \right) A\left( t \right)$ (Fig. 6a).

A possible kinetic law that captures the stretch-induced growth of the neurite is the Bingham-type relation

19 $$\begin{aligned} \frac{1}{\gamma }\frac{\text {d}{\gamma }}{\text {d}{t}} = k\left( \alpha - \alpha _\text {c} \right) _+ \end{aligned}$$

where *k* is a kinetic constant, and $\alpha _\text {c}\ge 1$ is a critical stretch above which growth may occur, as enforced by the ramp function $\left( \cdot \right) _+=\max \left( 0,\cdot \right)$ (Anthonisen and Grütter 2019; Goriely 2017). Below this threshold, deformation is purely elastic. For simplicity, we further assume that the neurite maintains a homeostatic cross-sectional area $A=A_0$ in the grown configuration, which implies $\gamma ^\perp =1$. Kinematically, $\dot{\gamma }/\gamma$ is a natural definition for the rate of growth, related to the *spatial velocity gradient* of continuum kinematics (Holzapfel 2000), and is the natural choice for growth processes that occur uniformly in a region. For growth processes that are restricted to a physical region the choice of $\dot{\gamma }$ rather than $\dot{\gamma }/\gamma$ in (19) would be a better modeling choice. Physically, (19) captures the *stretch-and-intercalation* process that has been proposed as a possible mechanism for bulk growth (Suter and Miller 2011; Holland et al. 2015), where the product $k\left( \alpha - \alpha _\text {c} \right) _+$ phenomenologically encompasses thermodynamical processes and cellular regulation pathways involved in intercalation which we ignore here. We note that similar laws have been used to describe the strain-induced growth of plant cell walls (Lockhart 1965; Boudon et al. 2015; Goriely et al. 2008; Ambrosi et al. 2019; Ali et al. 2014; Bozorg et al. 2016; Zhao et al. 2020).

Next, we need to specify the geometric constraint or load applied to the system. This choice is motivated by the experimental setting or biological scenario considered. Experimentally, various methods have been used to control either the length of the neurite (Bray 1984; Purohit and Smith 2016) or the applied tension (Dennerll et al. 1989; Heidemann et al. 1997). *In vivo*, we may assume that integrated neurites bound to the body are subject to an *imposed displacement*, insofar as their internal tension remains small and does not itself affect the growth of the embedding medium. In this case, $\lambda \left( t \right)$ is a prescribed function, and we may solve (19) using the identity $\alpha =\lambda /\gamma$:

20 $$\begin{aligned} \gamma \left( t \right) = {\text {e}}^{-t/\tau }+k \int ^t_0 \lambda \left( \xi \right) {\text {e}}^{-\left( t-\xi \right) /\tau }\,\text {d}{\xi } , \end{aligned}$$

where $\tau {:}{=}1/k\alpha _\text {c}$ defines a timescale for growth, and where we have taken $\gamma \left( 0 \right) =1$ and $\alpha >\alpha _\text {c}$.

For a ramped traction of the form $\lambda \left( t \right) = \lambda _0 + t/\tau '$, with $\lambda _0$ a constant and $\tau '$ a characteristic time of traction, (20) can be integrated to obtain

21 $$\begin{aligned} \gamma \left( t \right) = \frac{t}{\alpha _\text {c}\tau '}+\left( \frac{\lambda _0}{\alpha _\text {c}} - \frac{\tau }{\alpha _\text {c}\tau '} \right) + \left( 1 - \frac{\lambda _0}{\alpha _\text {c}}+\frac{\tau }{\alpha _\text {c}\tau '} \right) {\text {e}}^{-t/\tau }. \end{aligned}$$

In a *stretch-and-hold* experiment with $\lambda _0 \ge \alpha _\text {c}$ and $\tau ' \rightarrow \infty$, the axon is instantly stretched from its stress-free configuration $L_0$ to a fixed length $\lambda _0L_0$. In this case, the neurite relaxes exponentially (Dennerll et al. 1989), see Fig. 6c, and reaches a homeostatic stretch given by the critical yield stretch $\alpha _\text {c}$.

Alternatively, in a *speed-controlled* experiment, $\lambda _0=1$ with $\tau '$ finite, the axon is pulled with speed $L_0/\tau '$, as simulated in Fig. 6d. In this case, growth is asymptotically linear $\gamma \left( t \right) \sim t / \alpha _\text {c}\tau '$, and as before, the excess stretch $(\alpha -\alpha _\text {c})$ vanishes as $t\rightarrow \infty$. Note, however, that for $\alpha _\text {c} > 1$, the onset of growth is not at $t=0$ but is delayed to $t=\left( \alpha _\text {c}-1 \right) \tau '$.

Next, we consider a *force-controlled* experiment where the neurite is pulled with a given longitudinal external force *F*. In that case, we need to relate the stresses to the strains. To do so, we assume that, instantaneously, the material is hyperelastic and incompressible. It is then described via a Gibbs free energy density $\varPsi \left( \mathbf {A} \right)$ (in unit energy per intermediate volume). Incompressibility implies $\alpha ^\perp = 1/\sqrt{\alpha }$, from which we introduce the auxiliary energy function ${\widehat{\varPsi }}\left( \alpha \right) {:}{=}\varPsi \left( \alpha ,1/\sqrt{\alpha },1/\sqrt{\alpha } \right)$. In normal growth conditions, the timescale of elastic relaxation ($\sim$minute) is much shorter than that of irreversible expansion, which depends on relatively slow biophysical processes ($\sim$hour). Therefore, the system can be considered at quasi-static equilibrium at all times. The equilibrium condition follows from the principle of virtual work

22 $$\begin{aligned} \delta W = \delta W_\text {ext} + \delta W_\text {int}=0, \end{aligned}$$

where $\delta W_\text {ext} = F\delta \ell = FL\delta \alpha$ is the virtual work due to the traction force $F>0$, and $\delta W_\text {int} = -\delta ({LA{\widehat{\varPsi }}})$ is the virtual work due to the reaction force. Thus, the equilibrium configuration corresponds to a root of

23 $$\begin{aligned} F - A_0{\widehat{\varPsi }}'\left( \alpha \right) = 0. \end{aligned}$$

A variant of the stretch-and-hold experiment consists in pulling the neurite tip with a micro needle perpendicular to the neurite and whose base is fixed at a position $x=L_\text {n} \ge L_0$. Modeling the needle as a cantilever beam with length *D* and flexural rigidity *B*, we obtain the condition

24 $$\begin{aligned} \alpha = \frac{L_\text {n}}{L}\left[ 1 - \left( \frac{A_0D^3}{3BL_\text {n}} \right) {\widehat{\varPsi }}'\left( \alpha \right) \right] . \end{aligned}$$

Taking $B\rightarrow \infty$ gives the stretch-and-hold scenario seen before. However, the small deflection of the needle here provides a measure of the neurite tension, given by $3B/D^3 \left( L_\text {n} - \ell \right)$ (Zheng et al. 1991). Another popular experiment consists in pulling the neurite laterally (Bernal et al. 2007; Rajagopalan 2010; Dennerll et al. 1989). For the sake of brevity, we ignore this last case (see Goriely et al. 2015a, for details).

Lastly, we can also include the active properties of the neurite due to the actomyosin sheath that generates contractile forces. Several methods allow to model active effects in elasticity (Goriely 2018). For our problem, a possible approach consists in augmenting the balance equation (23) with an active stress $T_\text {a}<0$ so that

25 $$\begin{aligned} F - A_0 {\widehat{\varPsi }}'\left( \alpha \right) + A_0 T_\text {a} = 0. \end{aligned}$$

Thus, upon removal of the load, i.e., when $F=0$, the neurite is at equilibrium in a contracted configuration $\alpha <1$.

Several forms of the strain–energy function ${\widehat{\varPsi }}$ have been discussed in Goriely et al. (2015a). For example, we consider a neo-Hookean material with shear modulus $\mu$. The energy function is ${\widehat{\varPsi }}\left( \alpha \right) = \mu /2\left( \alpha ^2+2/\alpha -3 \right),$ and (23) reduces to the cubic $\alpha ^3-\left( {F}/{A_0\mu } \right) \alpha ^2-1=0$ which supports only one real solution for the stretch $\alpha$ entering (19). For a constant *F*, $\alpha$ is constant and growth is exponential.

In reality, towed neurites grow at a nearly constant speed depending on the applied force (Bray 1984), but this remains to be assessed on longer time scales. To explain this possible discrepancy, we stress that, in postulating (19), we assumed that growth takes place at *all* points of the shaft. The precise location of growth in stretched axons has not been fully elucidated (Futerman and Banker 1996; Smith 2009). For instance, in the compartmental model developed by Purohit and Smith (2016) (discussed more in details in Sect. 3.3), growth of stretched axons is assumed to take place via polymerization reactions localized in the axon hillock, i.e., the proximal segment of the neurite close to its junction with the soma. To describe this mechanism, we assume that growth happens only in a physical zone of constant length $L_\text {h}$ in the *intermediate* configuration, located near the proximal boundary, as shown in Fig. 6b. In this formulation, the hillock is *not* a Lagrangian material volume in the sense that its length changes with respect to the *reference* configuration $\varOmega _0$. We modify (19) to account for this new assumption, and we posit a *localized* exponential growth law:

26 $$\begin{aligned} \frac{1}{\gamma \left( X_0,t \right) }\frac{\partial {\gamma }}{\partial {t}}\left( X_0,t \right) = k\left( \alpha \left( t \right) \!-\! \alpha _\text {c} \right) _+{\mathcal {H}}\left[ L_\text {h} - X\left( X_0, t \right) \right] , \end{aligned}$$

where *X* depicts the axial coordinate in the intermediate configuration, and ${\mathcal {H}}$ is the Heaviside step function enforcing that growth only occurs for $X\le L_\text {h}$. Note that, in contrast to $\gamma$, $\alpha$ is still uniform along the whole shaft provided a uniformly-defined strain energy function. Thus, the resting length of the neurite $L\left( t \right)$ obeys

27 $$\begin{aligned} \frac{\text {d}{L}}{\text {d}{t}}&= \int _0^{L_0} \frac{\partial {\gamma }}{\partial {t}}\left( X_0,t \right) \,\text {d}{X_0} \nonumber \\&= \int _0^{L_0}\!\!\!\!k\left( \alpha \left( t \right) - \alpha _\text {c} \right) {\mathcal {H}}\left[ L_\text {h}\! -\! X\left( X_0, t \right) \right] \gamma \left( X_0,t \right) \,\text {d}{X_0}\nonumber \\&=\int _0^{L\left( t \right) } k\left( \alpha \left( t \right) - \alpha _\text {c} \right) {\mathcal {H}}\left( L_\text {h} - X \right) \,\text {d}{X}\nonumber \\&=L_\text {h} k\left( \alpha \left( t \right) - \alpha _\text {c} \right) , \end{aligned}$$

which is independent of $L\left( t \right)$. For a speed-controlled experiment, with pulling velocity ${\text {d}{\ell }}/{\text {d}{t}} = L_0/\tau '$, the elastic stretch tends to a small residual value of order

28 $$\begin{aligned} \alpha \sim \alpha _\text {c} +\frac{L_0\tau }{L_\text {h}\tau '}, \end{aligned}$$

for $t\gg \tau ' \gg \tau$, i.e., when pulling is slow; and becomes large,

29 $$\begin{aligned} \alpha \sim \sqrt{\alpha _\text {c}\frac{L_0\tau }{L_\text {h}\tau '}}, \end{aligned}$$

when $t\gg \tau \gg \tau '$, i.e., when pulling is fast, which may lead to disconnection. This is consistent with Purohit and Smith (2016) (see Sect. 3.3) in which axon tension plateaus at a finite value that depends on the pulling speed.

In a force-controlled experiment with tension *F*, the system develops a constant stretch $\alpha$ (23), and its size increases with constant speed ${\dot{\ell }} = L_\text {h}k \alpha \left( \alpha -\alpha _\text {c} \right)$. Although, locally, the growth mechanism itself is the same, this regime is very different from the exponential growth predicted by the previous growth model described by (19). This is due to the fact that the expanding zone $L_\text {h}$ remains constant (see Goriely 2017, pp. 69–70).

Interestingly, a popular growth law (Wang and Kuhl 2019; Holland et al. 2015; Goriely et al. 2015a) to describe stretched axons is

30 $$\begin{aligned} \frac{\text {d}{\gamma }}{\text {d}{t}} = k\left( \alpha -\alpha _\text {c} \right) _+. \end{aligned}$$

An example simulation for a speed-controlled experiment is shown in Fig. 6e. Since $\gamma$ is here uniform, the resting length increases as

31 $$\begin{aligned} \frac{\text {d}{L}}{\text {d}{t}} = L_0\frac{\text {d}{\gamma }}{\text {d}{t}} = L_0 k \left( \alpha -\alpha _\text {c} \right) _+, \end{aligned}$$

which is equivalent to (27) up to a rescaling. Thus, (30) *rheologically* captures the mechanical response of a neurite undergoing accretive or localized growth. Note, however, that, to our knowledge, there is no clear proof of the existence of such growth regime. In fact, every segment of the axon has the potential to undergo growth (Lamoureux et al. 2010). The hillock growth model proposed by Purohit and Smith (2016) is based on the observation that axons elongate mostly near the cell body, when the latter is towed, while the growth cone is held fixed with respect to the substrate (*reverse towing* experiment detailed in Lamoureux et al. 2010). However, this argument is not fully satisfactory, as Lamoureux et al. (2010) considered neurites embedded on a substrate, therefore not mechanically isolated. As shown in the next section, this second scenario is different, and, in this case, mechanics demands that the axon grows faster near the soma even when the whole shaft is assumed to expand. In conclusion, the exact location of growth in stretched axons and a correct morphoelastic representation for it remain an open problem.

#### One-dimensional models

The 0D models are suitable for neurites grown in controlled experimental conditions where longitudinal tension is homogeneous. However, in elongation mediated by the growth cone, this assumption will not hold due to the presence of dissipative forces such as cell-substrate adhesion, and one needs to consider variations of both stress and velocity along the neurite. In fact, it has been clearly shown that migrating presynaptic neurite stretch non-uniformly while elongating (Miller and Suter 2018; Athamneh et al. 2017; Lamoureux et al. 2010; O’Toole et al. 2008a).

**Fig. 7:**
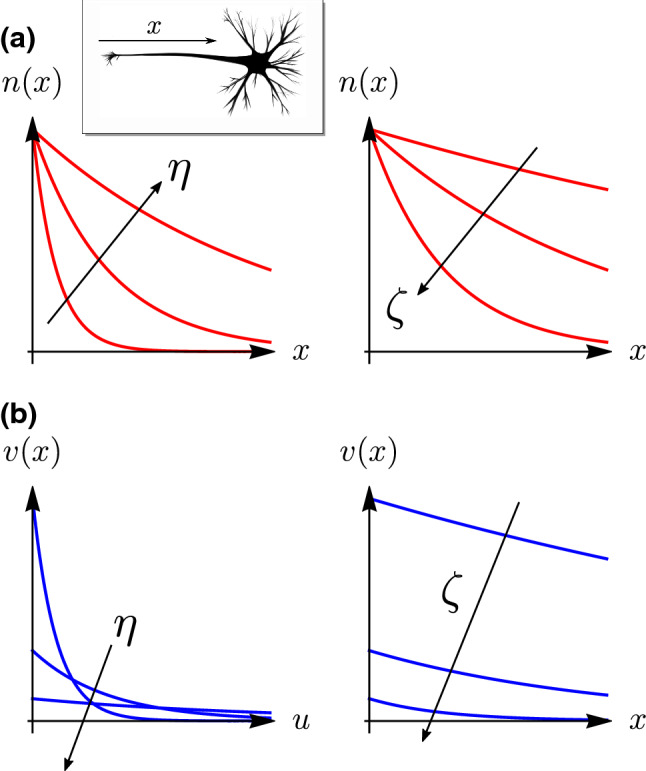
One-dimensional model of neurite growth. Effect of growth ($\eta$) and adhesion ($\zeta$) on **a** the tension profile *n*(*x*) versus *x* the distance from the growth cone and **b** the velocity profile *v*(*x*), obtained from (37). Inset shows the orientation of the *x*-axis w.r.t. the neurite

A simple and conceptually interesting model has been first introduced by O’Toole et al. (2008a) (and adapted to morphoelasticity by Goriely 2017, see pp. 80–85). In this model, the growing axon is seen as a simple fluid structure in contact with a substrate. Here, we focus on the permanent self-similar regime, for which the stress and velocity profile are constant in time in the vicinity of the neurite tip (Oliveri et al. 2021). Therefore, it is convenient to work in the advected frame attached to the tip, and to parameterize the neurite by the coordinate *x* oriented toward the soma and originated at the neurite tip (Fig. 7, inset).

At any position *x*, we may virtually cut the neurite to define $n\left( x,t \right)$ the force applied by the cross section $x^+$ to the cross section $x^-$. The classic procedure to derive the local balance equation based on the balance of linear momentum (Goriely 2017) provides an equation for *n*:

32 $$\begin{aligned} \frac{\text {d}{n}}{\text {d}{x}} + f\left( x \right) =0, \end{aligned}$$

where *f* depicts the lineal density of tangential body force applied along the shaft. In order to model the interaction between the shaft and the substrate, we assume a body force due to damping of the form

33 $$\begin{aligned} f\left( x \right) = \zeta v\left( x \right) , \end{aligned}$$

with *v* the anterograde velocity in the laboratory frame, and $\zeta$ a friction coefficient. In addition, we postulate the constitutive relation

34 $$\begin{aligned} \frac{\text {d}{v}}{\text {d}{x}} = -\frac{n}{A\eta }, \end{aligned}$$

expressing the stress-induced growth of the shaft, with $\eta$ a bulk growth parameter analogous to a viscosity. This constitutive law is akin to the exponential growth law seen previously (Sect. 3.2.1) where $\eta$ plays a role similar to $\mu /k$. Note that the minus sign results from the choice of orientation for the *x*-axis, opposite to the anterograde motion.

On differentiating (32) and plugging (33, 34), we obtain:

35 $$\begin{aligned} \frac{\text {d}^{2}{n}}{\text {d}{x}^{2}}- \frac{n\left( x \right) }{h^2} = 0, \end{aligned}$$

where $h = \sqrt{A\eta /\zeta }$ defines a characteristic length for the problem. The distal end ($x=0$) of the neurite is subject to a tensile load *F*, while tension vanishes for $x \gg h$, as energy dissipates through friction. This dictates the boundary conditions

36 $$\begin{aligned} {\left. n \right| _{\ell \left( t \right) ,t}} = F ,\quad {\left. n \right| _{\infty ,t}}=0 , \end{aligned}$$

and the solution for the tension and the velocity profiles

37 $$\begin{aligned} n\left( x \right) = F e^{-x/h},\quad v\left( x \right) =\frac{Fe^{-x/h}}{\sqrt{A\eta \zeta }}, \end{aligned}$$

shown in Fig. 7, which illustrates the effects of both parameters $\eta$ and $\zeta$. We have in particular the two limits

38 $$\begin{aligned} \lim _{\eta \rightarrow \infty } v\left( x \right) = 0, \quad \lim _{\eta \rightarrow 0} v\left( x \right) = {\left\{ \begin{array}{ll} 0 &{}\quad \text {if}\quad x>0\\ \infty &{}\quad \text {if}\quad x=0 \end{array}\right. } \end{aligned}$$

that, respectively, correspond to a stalling regime, when neurite viscosity is high, and a rupture regime, when viscosity is low, with a strain at the tip ${\text {d}{v}}/{\text {d}{x}}\rightarrow \infty$. The adhesion $\zeta$ both reduces the velocity of the neurite and the propagation of forces along the shaft. We stress that the tip speed $v\left( \ell \left( t \right) ,t \right) =F/\sqrt{A\zeta \eta }$ is constant in time since growth happens in an effectively finite zone *a*, as discussed in Sect. 3.2.1, which for *a* small, results effectively in a tip growth regime. Experimentally, the tension profile can be measured from the spatial variation of the cross-sectional area. Indeed, assuming an incompressible neurite, we compute

39 $$\begin{aligned} a \left( x \right) = A \left( 1+\frac{F}{EA} e^{-x/h} \right) ^{-1}. \end{aligned}$$

**Fig. 8:**
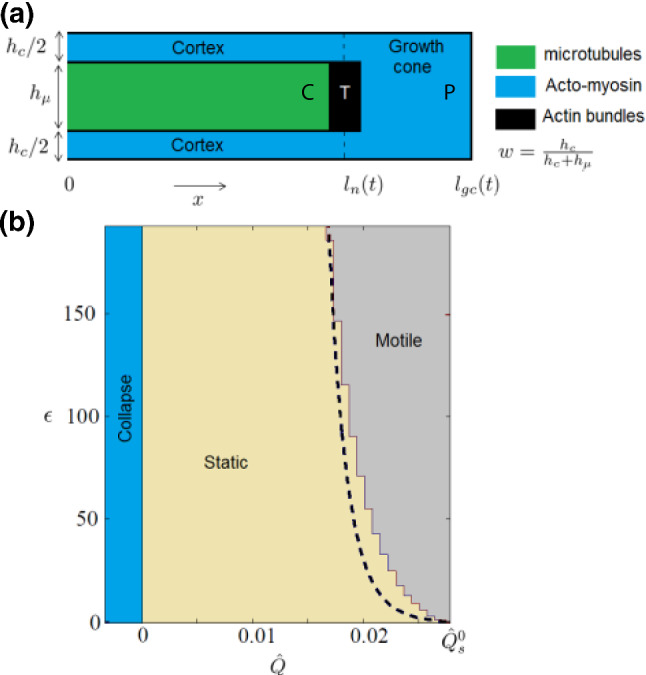
Multicompartmental mechanical modeling of neurite growth, adapted from Recho et al. 2016 with permission from the *American Physical Society*. **a** The neurite is represented as three connected compartments: the growing microtubule core, a contractile actomyosin sheath and the growth cone. **b** Parameter space $\epsilon$ versus ${\widehat{Q}}$, where $\epsilon$ measures the ratio between the actomyosin viscosity and the effective microtubule core viscosity, and ${\widehat{Q}}$ measures the excess of growth cone traction with respect to actomyosin contraction. We clearly see that growth is possible only if traction is sufficiently larger than contraction. Dashed line is an analytical estimate of the boundary between the collapse and motile states, obtained from a simplification of the model

An extension of this model was developed by Recho et al. (2016) using a similar formalism. In this extended model, the neurite is composed of three compartments, shown in Fig. 8a. The microtubule core is represented by an elastic solid undergoing mass depletion and mass addition that aim to maintain a density at chemical equilibrium. Namely, a compressed core will have an excess density, compensated by a mass removal. Conversely, mass addition allows to compensate for the decrease in density due to an excessive traction. This mechanism is coupled with an elastic constitutive behavior that provides the pressure of the core. The core is itself embedded inside a *viscocontractile* actomyosin cortex. The two compartments are mechanically coupled via frictional interactions. A no-flux boundary condition at the junction between the microtubule core and the growth cone implies the absence of tip growth. The structure formed by these two compartments is pulled at its distal end by a third compartment that represents the advancing growth cone made up of actomyosin and pulling on the substrate. Finally, the dynamics of the structure is obtained from first physical principles, namely the balance of mass and the balance of momentum.

The resulting model supports three states, shown in Fig. 8b. A retraction regime occurs when microtubule pressure and growth cone traction are insufficient to overcome the actomyosin contractile forces. Conversely, relatively weak contractility reduces the compression along the microtubules and facilitates elongation. Due to the combination of several threshold effects, the model also predicts the possibility of stalling, i.e., a situation where the structure is static. In this state, the structure neither grows nor retracts unless the parameters promoting either regimes exceed a threshold value. This scenario is reminiscent of the seminal “rubber band gun” model proposed by Buxbaum and Heidemann (1988), who considered microtubules subject to longitudinal compression due to the front membrane of the neurite. Traction forces produced by the growth cone reduce compression, decreasing the free energy required for microtubule assembly and growth.

The power of Recho et al.’s model is that it addresses each structural compartment of the neurite separately and relates their interactions to parameters that can be modified experimentally. Indeed, it is able to reproduce in details the observed qualitative effects induced by drugs, such as blebbistatin, that reduces myosin II motor contractility; cytochalasin, that inhibits actin polymerization; trypsin, that reduces substrate adhesion; nocodazole, that destabilizes microtubules; or taxol, that stabilizes microtubules. These drugs affect distinct and well-identified target parameters of the models, and their respective effects can be simulated specifically. This also explains coupled effects that can appear as paradoxical when looked at in isolation.

The models presented so far are based upon continuum formulations, which allows us to make use of well-known mathematical techniques in order to derive analytical predictions, and extend these predictions numerically. Note that the neurite cytoplasm is in reality composed of several polymers that interact and contribute to global mechanical, geometrical and dynamical properties in a non-trivial way. Thus, continuum models are an approximation that must be used with proper caution, and that may be unsuited to representing subtle small-scale properties and processes. To capture molecular details and their emergent properties, many authors have recently examined neurites at a discrete level, for instance, by representing microtubules as discrete structures connected via cross-linking proteins (Montanino and Kleiven 2018; Ahmadzadeh et al. 2012, 2015; Peter and Mofrad 2012; Jakobs et al. 2015, 2020; De Rooij et al. 2017; De Rooij and Kuhl 2018a; De Rooij et al. 2018; De Rooij and Kuhl 2018b). This sophisticated and realistic type of approach comes naturally with increased model complexity and computational difficulty, but allows for addressing finer questions on the effects of particular proteins or specific mechanisms.

### Coupling mechanics and transport

Mechanics and transport are two coupled facets of neurite growth. The classic theory of transport-mediated growth assumes that neurite elongation is controlled by the concentration of material, supplied through a production rate that is generally taken to be constant. Clearly, this view cannot capture scenarios where axon length is controlled externally and not by tubulin concentration, like in stretch growth. In contrast, mechanical models describe the expansion rate as a rheological process akin to plasticity and involve the internal tension of the neurite as the main growth determinant. However, as a neurite elongates and thickens, it naturally increases its demand in material (O’Toole et al. 2008b). In particular, to sustain rapid elongation and adapt to changes in stretch rates, the cell body potentially actively modulates the production and transport of material to support growth and avoid disconnection or injury (Suter and Miller 2011; Athamneh and Suter 2015); however, little is known on this mechanism. For instance, Ahmed and Saif (2014) showed that increased tension correlates with faster vesicle transport, whereas Loverde et al. (2011) reported reduced fast axonal transport due to strain. Mechanisms underlying a stretch-mediated regulation of transport remain elusive and might involve mechanosensitive ion channels (Franze 2020); however, this has not been demonstrated systematically. Generally speaking, the rheology of neurites is a complex and dynamically regulated process that is not completely captured by passive mechanical models with constant kinetic properties. For example, it is plausible that parameters such as *k* in (19), or $\eta$ in (34) will depend on material availability, especially in long and fast-stretching neurites.

In contrast to the models detailed in Section 3.1.2 that focus on tip growth, O’Toole and Miller (2011); O’Toole et al. (2008b) investigate the contribution of stretching in axonal transport of mitochondria. Here, we consider an isolated axon fixed at its base and stretched with speed *v*, with length $\ell \left( t \right) =\ell _0+vt$. It can be experimentally observed that mitochondrial density is more or less uniform along the axon and increases with rate $\alpha$ as time progresses. Thus, we assume a lineal density of the form $c\left( x,t \right) = c_0 + \alpha t$. From the balance of mass, we obtain the flux along the axon:

40 $$\begin{aligned} J\left( x,t \right) = - x \left( \frac{c_0 + \alpha t}{\tau }+\alpha \right) + J_0\left( t \right) , \end{aligned}$$

where $\ln \left( 2 \right) \times \tau$ defines the half-life of a mitochondrion, and $J_0$ is the flux entering the axon at $x=0$. A no-flux boundary condition $J\left( \ell \left( t \right) ,t \right) =vc\left( \ell \left( t \right) ,t \right)$ at the tip provides

41 $$\begin{aligned} J_0 \left( t \right) = \frac{\left( c_0 + \alpha t \right) \left( \ell _0 + v t \right) }{\tau } + v\left( c_0+\alpha t \right) + \alpha \left( \ell _0 + v t \right) , \end{aligned}$$

that grows quadratically in time. Along the axon, stretch results in a purely advective flux, which under uniform deformation is given by $J_\text {stretch}\left( x,t \right) = xvc\left( t \right) /\ell \left( t \right)$. Thus, the rest of the flux $J_\text {other}\left( x,t \right) = J\left( x,t \right) - J_\text {stretch}\left( x,t \right)$, due to cellular transport, decreases linearly along the axon as

42 $$\begin{aligned} J_\text {other} \left( x,t \right) = \left( 1 - \frac{x}{\ell _0 + vt} \right) J_0 \left( t \right) . \end{aligned}$$

**Fig. 9:**
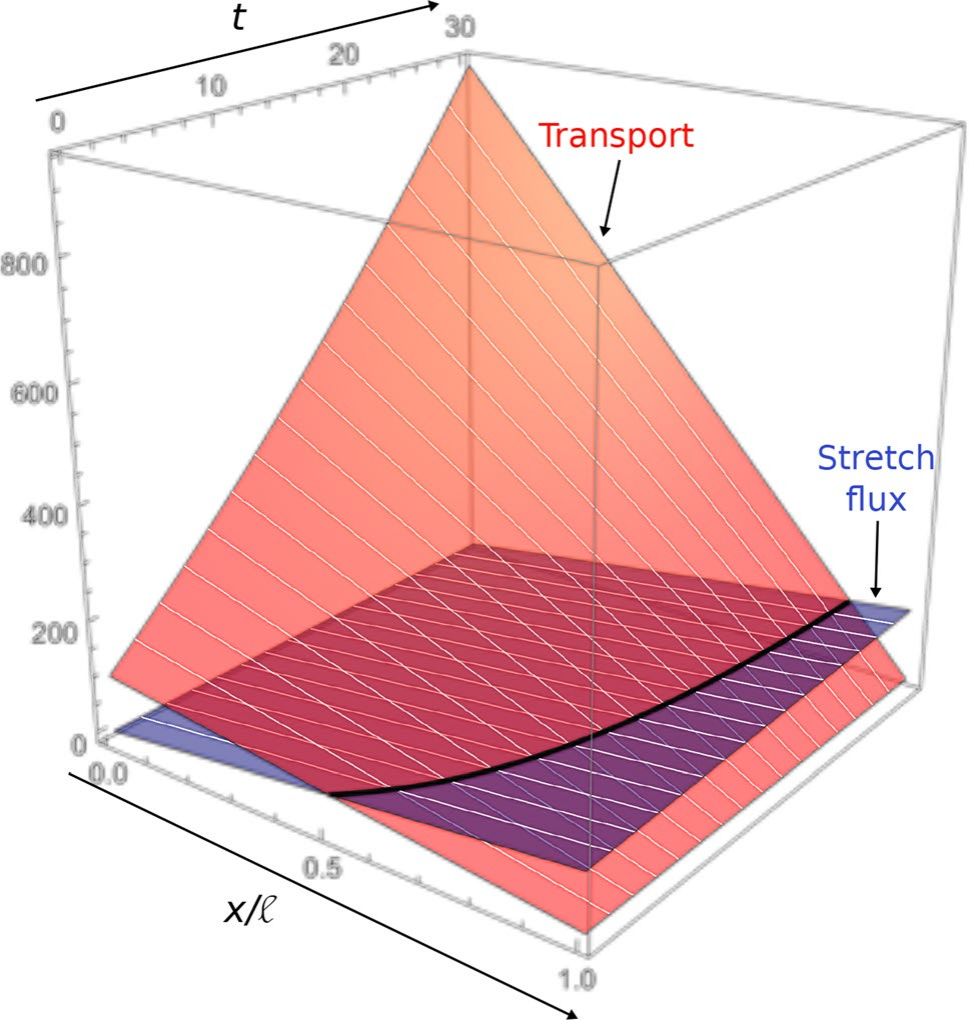
Flux due to stretch $J_\text {stretch}$ and to other contributions $J_\text {other}$ (mainly transport) versus time *t* and normalized abscissa $x/\ell \left( t \right)$ obtained from (40–42) (O’Toole et al. 2008b; O’Toole and Miller 2011)

Figure 9 shows the spatial temporal evolution of both $J_\text {stretch}$ and $J_\text {other}$. Note that the authors also address the case where the axon is attached to its substrate via adhesion (O’Toole et al. 2008a), in which case $J_\text {other}$ becomes the dominating contribution to the flux (since advection is localized at the tip, see Sect. 3.2.2).

By contrast to classic 1D models of transport (Sect. 3.1.2), the cellular contribution to the flux here decreases along the axon and depends on *v* and $\ell$ in a non-trivial way. This strongly suggests the existence of a mechanism allowing the axon to sense its own length and elongation rate, in order to achieve uniform mitochondrial density during stretch growth. However, this approach, which consists in weighing the various contributions to the flux using balance arguments, does not allow us to precisely infer the mechanisms involved.

**Fig. 10:**
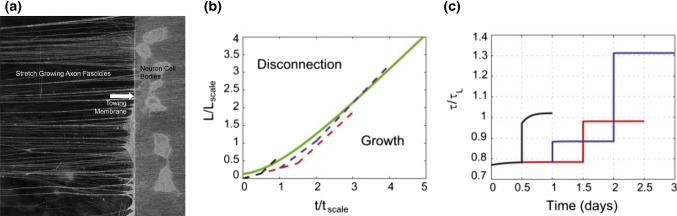
**a** Two populations of neurons are plated on two adjacent substrates (swine dorsal root ganglia neurons). Over a few days, axons sprout and connect the two neuron populations. A microstepper motor system then pulls the towing membrane away from the adjacent substrate at accelerating rates. One population shown on the towing platform (right) and growing axons (left). **b**–**c** Purohit and Smith’s model of growth and disconnection of stretched axons. **b** The green line shows the limiting $\ell \left( t \right)$ curve above which the axon disconnects. Dashed lines show different stretching trajectories with piecewise constant growth rate. **c** Corresponding tension $\tau$ normalized by failure tension $\tau _L$. The black and blue curves illustrate two different disconnection scenarios associated with different traction parameters. Images reproduced from Purohit and Smith (2016) with permission from Elsevier

To model mechanotransduction and growth in axons, Purohit and Smith (2016) reexamined Samuels et al.’s model to address the case of integrated axons subject to externally applied stretch, coupling mechanics to transport via a hypothesized stretch-activated ion entry (Fig. 10a). Here, the pulling speed is imposed, and therefore, transport processes mostly contribute to changing the internal state of the neurite, i.e., its cross-sectional area and tension. As in Samuels et al. (1996) and (13), the transport rate is assumed to be proportional to growth speed. To predict disconnection, an increase in tubulin production at the soma as a response to ion entry is hypothesized. The latter is promoted by axon tension, which decreases the free energy required for opening mechanosensitive ion channels along the shaft. The entry of ions in turn triggers an increase in cellular material production rate (*S* with our notation) which models the response of the axon to fast stretching. Finally, the resulting tension is compared with a critical disconnection tension, as shown in Fig. 10b, c, which allows to quantitatively evaluate the risk of nerve rupture, which is of interest, for instance, in the design of regeneration procedures.

In the last few decades, the active regulation of growth and cellular processes by mechanical forces has become a landmark of developmental biology (Hamant 2017). From a modeling point of view, the nonlinear coupling between forces and geometry can shed light on rich and non-intuitive behaviors. In neurons, this type of regulation remains poorly understood. Arguably, the development of mathematical models integrating active properties such as mechanosensing will be instrumental to building a unified theory of neurite growth.

## Modeling the neurites in their environment

We have discussed models of neurites growing along a line with a focus on the fundamental mechanisms of elongation. However, the path and pattern formation of dendrites and axons growing in 2D or 3D is also of fundamental importance to understand the development of the brain architecture. Thus, we next describe the morphogenesis of neurons embedded in the multidimensional space and in relationship with their physical environment.

### Guidance

**Fig. 11:**
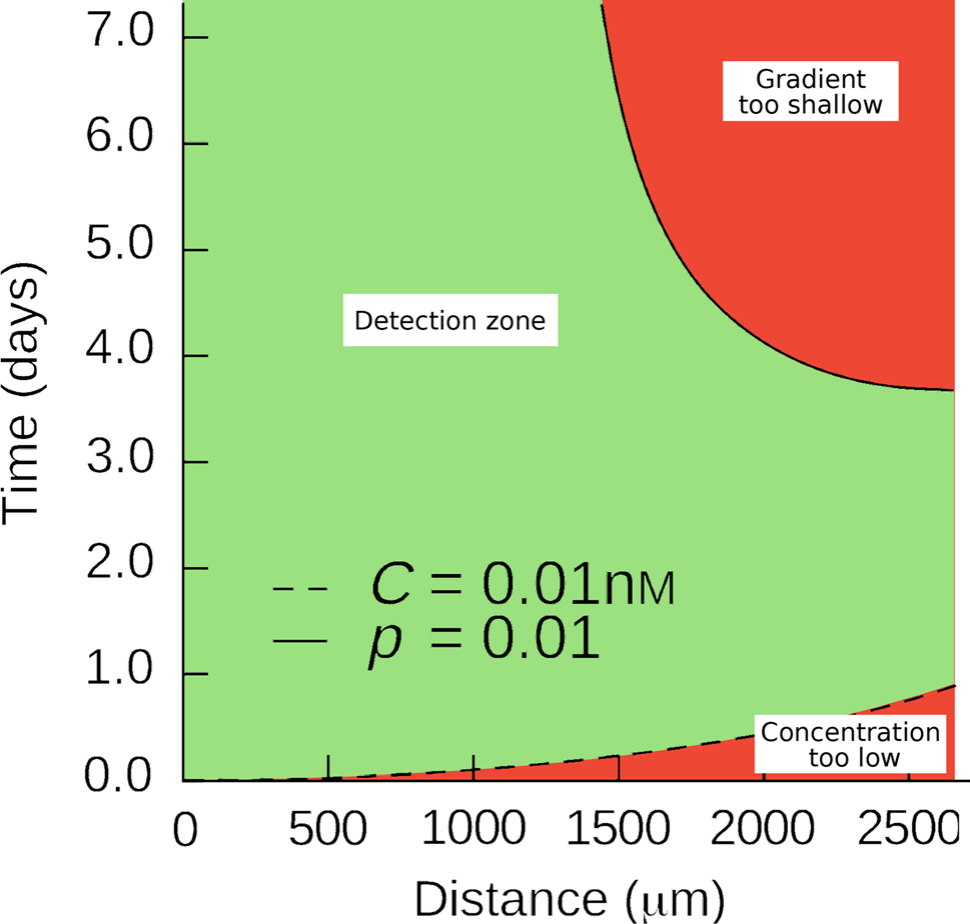
Chemotactic detection zone based on Goodhill (1998). Parameter values are carefully discussed in the original article. For a given distance from the source, we compute the time interval during which detection is possible, represented by the green domain. The red domain represents the times when chemotaxis is not possible yet due to insufficient ligand concentration (minimum concentration *C*) or no longer possible due to a shallow gradient (constraint on *p*). For the parameter values used by the authors, the upper constraint on the concentration (saturated receptors) concerns points that are very close to the origin and is therefore ignored here. We see that for $r \lesssim 1500\,{\upmu }\hbox {m}$, detection is always possible after a given time. Image adapted from Goodhill (1998) (with permission from *Elsevier*)

During guidance, the axon tip perceives and transduces information from its local environment to find its path. Historically, our understanding of this process was chiefly predicated upon chemical signaling, in particular chemotaxis (Mortimer et al. 2008), which is the ubiquitous and predominant modality of growth cone pathfinding. For chemotactic information to be exploitable by a cell, a number of physical constraints must be simultaneously satisfied (see the classic article by Berg and Purcell (1977)). In a sequel of papers, Goodhill and coworkers developed seminal ideas to address the constraints of growth cone chemotaxis, using arguments from diffusion theory (Goodhill 1997; Goodhill and Urbach 2003; Goodhill 1998; Goodhill and Baier 1998; Goodhill and Urbach 1999). A core idea is that for a difference $\varDelta C$ in chemical concentration *C* to be detectable by a growth cone across its length scale $\varDelta r$, several conditions must be simultaneously satisfied. First, the overall concentration must not be too high (saturated receptors) or too low (insufficient binding) (Goodhill 1998). Second, the gradient itself, e.g., the fractional difference $p=\varDelta C/C\approx \left( \varDelta r / C \right) \,{\partial {C}}/{\partial {r}}$, must be sufficiently large to overcome noise and provide an informative signal. The concentration *C* is obtained by solving the diffusion profile associated with a point source of ligand. It is well known that there is no physical (positive) solution to the diffusion equation in one or two dimensions, which can be alleviated by taking into account the natural extinction of the ligand (Krottje and Van Ooyen 2007). However, in three dimensions, a ligand diffusing from a steady point source has a concentration *C* given by

43 $$\begin{aligned} C\left( r, t \right) = \frac{q}{4\pi D r}{{\,\mathrm{erfc}\,}}{\frac{r}{\sqrt{4Dt}}}, \end{aligned}$$

with *r* the distance from the source; ${{\,\mathrm{erfc}\,}}$ the complementary error function; *D* the diffusion coefficient; and *q* the rate of ligand production at the source. Therefore, combining (43) with the constraints on detection provides the range of radii $r\in \left[ r_\text {min}\left( t \right) ,r_\text {max}\left( t \right) \right]$ within which detection is possible at a given time *t*, as shown in Fig. 11.

While chemotaxis is the most studied and main modality of guidance, other less conspicuous guidance mechanisms also are at play. For example, there is now considerable evidence that the mechanical stiffness of the tissues is used as a regulatory cue during the formation of the nervous system (Abuwarda and Pathak 2020; Koch et al. 2012; Chan and Odde 2008; Koser et al. 2016; Franze 2020). Chan and Odde (2008) proposed a stochastic model of growth cone locomotion, modeling the actin motor-clutch system at the edge of the growth cone, based on attachment and breakage of focal adhesion bonds depending on substrate stiffness (see also the mean-field treatment by Bangasser and Odde 2013). The authors predicted that the growth cone traction, which depends on its ability to pull its substrate, depends on medium compliance in a nonlinear fashion. On soft substrates, the growth cone develops traction by a so-called *load-and-fail* dynamics where the adhesion is maintained for a sufficient amount of time. However, on excessively stiff media the same mechanism results in a catastrophic loss of adhesion leading to a *frictional slippage* regime, in which the growth cone cannot progress efficiently.

While these works discuss mechanisms and constraints at play in chemotaxis or mechanical guidance, they do not seek to describe the path of the growth cone as a response to these stimuli. To that end, trajectories of growth cones seen as a random motion on a 2D substrate have been studied by multiple authors (Katz et al. 1984; Mortimer et al. 2010; Maskery et al. 2004; Hentschel and Van Ooyen 1999, 2000; Krottje and Van Ooyen 2007; Borisyuk et al. 2008; Buettner et al. 1994; Wang et al. 2003; Pearson et al. 2011; Segev and Ben-Jacob 2000, 2001; Basso et al. 2019; Yurchenko et al. 2019, 2021; Davis et al. 2017; Roberts et al. 2014); see also the review by Maskery and Shinbrot (2005). The main focus in these works is not the mechanism of elongation, as treated before, but rather the shape and statistical properties of growth cone trajectories, as a function of external cues.

A simple model for the motion of a growth cone $\mathbf {P}\left( t \right)$ consists in considering the competition between stochastic, random walk events, and deterministic events based on guidance cues. Within this framework, a general probabilistic process describing growth cone motion is

44 $$\begin{aligned} \text {d}{\mathbf {P}} = \mathbf {V}\left( t \right) \,\text {d}{t}+\sqrt{D}\,\text {d}{\mathbf {W}}, \end{aligned}$$

where $\mathbf {V}$ is a deterministic drift that depends on sensory cues; $\mathbf {W}$ is the Wiener stochastic process; and *D* is a diffusion constant (Maskery and Shinbrot 2005). For example, the case where $\mathbf {V}$ is constant (e.g., when the growth cone perceives a constant chemical cue) can be decomposed as (i) a translation $\mathbf {V}t$ combined with (ii) a diffusion with typical travel length $\sqrt{Dt}$. Note that (44) is not specific to growth cones and in particular does not capture the directional persistence of growing neurites (Katz 1985). An alternative to (44) is the Langevin equation for Brownian motion that includes inertial forces which can mimic this effect. Curvature effects can also be included by assuming that guidance cues affect the direction of the tip, rather than its position, which allows to dampen abrupt turns (Krottje and Van Ooyen 2007; Borisyuk et al. 2008; Pearson et al. 2011).

To model chemotaxis, Hentschel and Van Ooyen (1999), for instance, considered a version of (44) including multiple chemical agents $\mu$, and assuming that growth cones follow the gradient of each concentration field $c_\mu$ as

45 $$\begin{aligned} \mathbf {V} \left( t \right) = \sum _{\mu } \lambda _{\mu } \nabla c_{\mu } \left[ \mathbf {P}\left( t \right) , t \right] , \end{aligned}$$

where the coefficients $\lambda _{\mu }$ quantify the strength of the response elicited by each agent $\mu$, which can be either repulsive, $\lambda _{\mu }<0$, or attractive with $\lambda _{\mu }>0$. Mathematically, the chemotactic force $\mathbf {V}$ assumes a conservative structure where the terms $\left( -\lambda _{\mu } c_{\mu } \right)$ relate to chemotactic potentials.

These previous approaches are simple and relatively easy to compare with experiments. However, they are rudimentary from the point of view of cellular mechanisms. Several authors have represented the cellular effects involved in growth cone motion by using more detailed representations of the growth cone sensory and locomotory apparatus. For instance, Aletti et al. (2008); Goodhill et al. (2004); Xu et al. (2005) represent the growth cone as a semicircle covered with binding sites. These sites are engaged with a probability that depends on the local value of ligand concentration that may vary across the growth cone diameter. The new orientation of the growth cone is then modified according to the direction of maximum binding. An extended model accounting for locomotion was used by Betz et al. (2009) who also represented the random fluctuations of the growth cone edge governed by a Langevin-like dynamics controlled by ligand binding, from which they deduce growth cone trajectory.

Haptotactic guidance has been addressed as well, for instance, by Van Veen and Van Pelt (1992), who modeled the contacts between the growth cone and discrete adhesive loci. When one adhesion site is detected within the growth cone, a force is generated and redirects the growth cone toward it. A similar approach was also employed by Li et al. (1995).

**Fig. 12:**
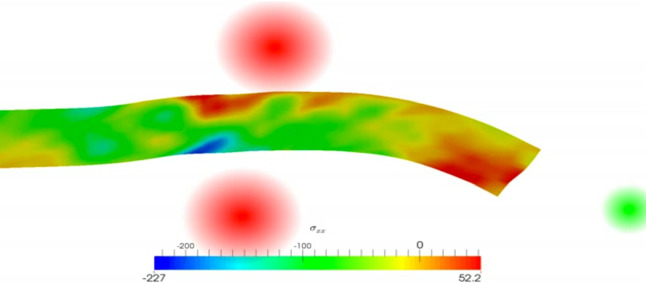
A simulation of axon growth and chemotactic guidance (the red fields denotes repellent and the green one the attractant). The color within the axon shaft gives the $\sigma _{xx}$ component of the stress within the structure, the *x*-axis being along the horizontal (based on the method given in García-Grajales et al. 2017)

Aeschlimann and Tettoni made a step toward an integrated mechanistic approach of chemotaxis and proposed a feedback mechanism where a chemical gradient is first detected by the growth cone filopodia, which in turn triggers the entry of calcium ions at their base (Aeschlimann 2000; Aeschlimann and Tettoni 2001). The influx and diffusion of calcium along the growth cone periphery stimulate further protrusion of filopodia. (More complex computational models for calcium signaling have also been investigated, see Roccasalvo et al. 2015; Forbes et al. 2012; Sutherland et al. 2014; Hely et al. 1998.) The feedback loop proposed by Aeschlimann and Tettoni is combined with a mechanical model for filopodium retraction. This retraction produces a resultant that pulls the growth cone toward the stimulus. However, to actually operate a turn, the growth cone must bend the microtubule bundle (Franze 2020). To model this effect, the authors represent the trailing shaft as a chain of viscoelastic springs connected by angular springs. (This approach may be seen as the discretization of a morphoelastic rod, see Moulton et al. 2013.) In line with Aeschlimann and Tettoni’s model of the morphoviscoelastic properties of the shaft, Zubler and Douglas (2009) proposed a detailed computational models of neurite migration in 3D, which includes steric hindrance due to other cells. Recently, García-Grajales et al. (2017) also proposed a cell-level morphoelastic approach by extending Recho et al. (2016) to a computational framework. In this approach, the authors use a multidimensional model of the shaft, including differential growth, contraction, bending, torsion or shear, and an advanced finite-element method is proposed for simulations over long distances, as shown in Fig. 12.

Overall, these models for axon growth and guidance describe, with various level of details, the behavior of a single axon in response to a stimulus field. They can be linked to studies of the growth cone to understand how a signal is sampled and integrated. Independently of these microscopic mechanisms, the overall macroscopic behavior of single axons is very much as expected. However, in many settings axons tend to interact and bundle together. Hence modeling these interactions is key to understand the formation of neuronal networks.

### Collective migration and fasciculation

It is observed that axons often migrate and bundle together in a process called *fasciculation* which is believed to improve the accuracy of axonal projections toward their target (Van Vactor 1998). An initial fasciculation is often terminated by defasciculation to innervate the target zone and establish precise connections. Theoretically, this process of fasciculation and defasciculation is an example of collective behavior where different biological units come together and interact, creating in the process emergent structures and non-intuitive dynamics (Vicsek and Zafeiris 2012).

**Fig. 13:**
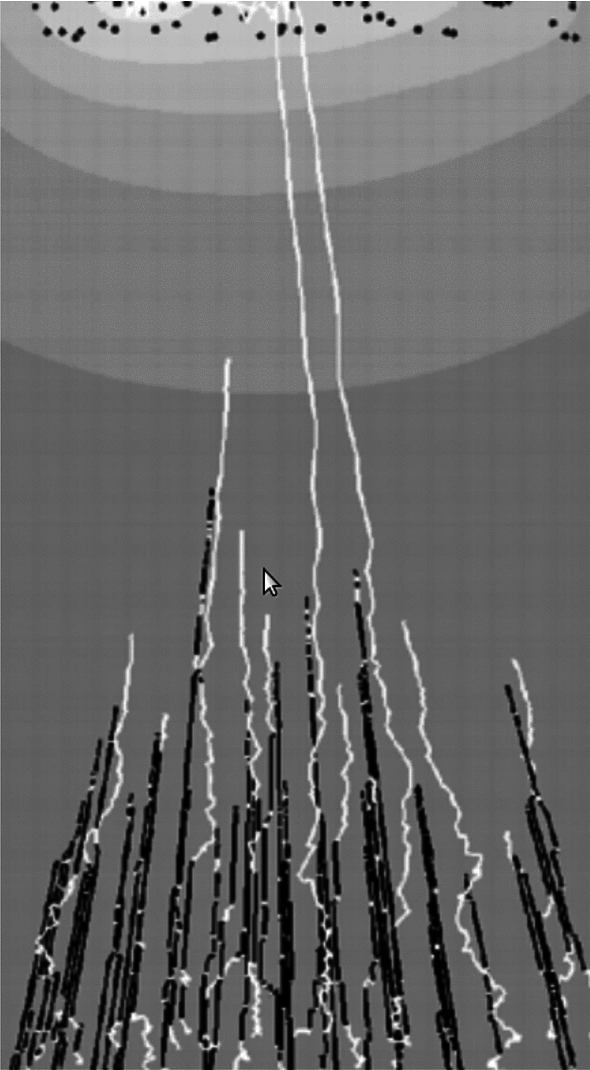
Initial axon development in the presence of contact attraction, simulated by Hentschel and Van Ooyen (1999). The axons are originated in the bottom boundary of the domain, and then, they migrate to the targets distributed along the top boundary. The axons are responding to the chemoattractant gradient setup by the layer of target cells and are subject to random movements. (Lighter regions show higher concentration.) The bundled axons (in black) move in a less random manner and grow slower than the unbundled ones (in white). Note the development of unbundled pioneer axons. Image reproduced from Hentschel and Van Ooyen (1999) with permission from the *Royal Society*

The Hentschel–Van Ooyen model given by equations (44–45) can be extended to include collective effects by considering growth-cone-secreted ligands, namely, chemical fields $c_i$ generated by multiple moving growth cones $\mathbf {P}_i\left( t \right)$. Unsurprisingly, the net effect is the formation of densely packed bundles. Those can separate in response to a new regulatory cue secreted by the targets, and instructing defasciculation. Note that the authors ignored the transient effects due to diffusion of ligands from a moving source and focused on quasi-steady regimes. (Non-steady solutions were studied numerically by Krottje and Van Ooyen (2007).) In another version of the model, the authors introduce fasciculation due to short-range contacts combined with noise in the growth cone trajectories. Interestingly, this accounts for the emergence of so-called *pioneer axons* (Fig. 13), namely axons that grow first toward a target region, and that have been proposed to lead the way for the rest of the bundle. This behavior can be explained by the fact that, in the model, unbundled axons show more random motions and therefore explore more of their surroundings. Therefore, those axons are more likely to reach areas of the domain where the chemoattractant gradient is steeper and chemoattraction is stronger, which leads them to further venture ahead of the group and migrate on their own. The track formed by pioneer growth cones is then followed by the rest of the bundle.

Analytical predictions are difficult to derive from these off-lattice multiagent models. By simplifying the geometry of the problem using a discrete random walk approach, Chaudhuri et al. (2011) examined the statistical properties of fasciculation. In their model, individual growth cones progress along the edges of a rhombic grid. At each discrete step of the automaton, each growth cone makes one step on the lattice and steers right or left according to a probability that depends on the presence of neighboring axons governing random fasciculation and defasciculation events. This model does not include chemotactic signals and focuses on axon–axon interactions. However, it includes other interesting processes such as the removal of existing axons with a given typical lifetime, or the difference in bundling affinities between two types of axon with different chemical signatures, resulting in sorting dynamics. This approach allows for the use of mean-field arguments, combined with relatively straightforward numerical simulations, to build insight into the non-equilibrium statistical mechanics aspects of fasciculation.

**Fig. 14:**
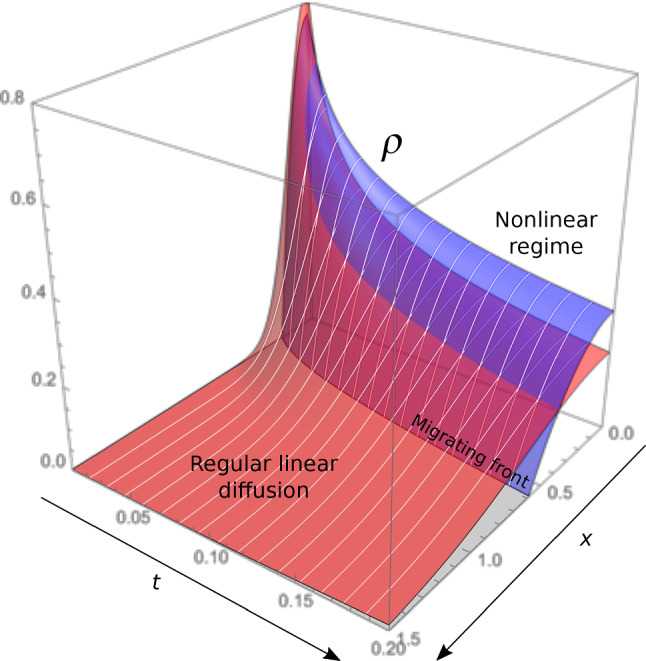
Diffusion of a population of growth cones obtained from (46) (De Gennes 2007). We show two types of exact similarity solution, respectively, associated with the linear regime (red) where regular diffusion ($D_0$) dominates, and in the idealized strong nonlinear regime (blue) where diffusion is small compared to growth-cone self-repulsion ($C\lambda _c\rho$). In the second scenario, a sharp linear front of slope $\sim t^{-2/3}$ advances slowly like $\sim t^{1/3}$, whereas in the regular diffusion, the front flattens like $\sim \exp \left( -x^2/4t \right) /\sqrt{t}$ with a typical band width of order $\sim t^{1/2}$

Another classic approach to collective cell migration and chemotaxis consists in assuming that a population of cells can be modeled as a continuous entity, which naturally lends itself to a continuum treatment in terms of partial differential equations (Murray 1993; Painter 2019). This approach has been employed to study the formation of neural crest (Giniūnaité et al. 2020), angiogenesis (Pillay et al. 2017) and epidermal wound healing (Sherratt and Murray 1990), for instance. Surprisingly, it has not been applied systematically to nerve cells, arguably due to their large and peculiar morphological structure that differs from most other cells. In a relatively unnoticed paper, the late Nobel Prize laureate P.-G. de Gennes proposed a simple mean-field model for collective axon migration (essentially in 1D), where a continuous population of growth cones is described by a density $\rho$ (De Gennes 2007). The flux *J* encompasses the isotropic random motion of growth cones, which is analogous to diffusion (with constant $D_0$), and a chemotactic flux $J_\text {chemo}$ (Murray 1993). In particular, the author postulates that a chemorepellent *c* is itself secreted by the growth cones. Upon fast ligand diffusion and decay, he assumes that $c = C\rho$ with *C* a constant. Thus, assuming that growth cones are repelled with velocity $v = -\lambda _c{\partial {c}}/{\partial {x}}$ as in (45), with $\lambda _c>0$, one derives the advective flux $J_\text {chemo} = - C\lambda _c\rho {\partial {\rho }}/{\partial {x}}$. The standard continuity equation

46 $$\begin{aligned} \frac{\partial {\rho }}{\partial {t}} + \frac{\partial {}}{\partial {x}}\left[ D\left( \rho \right) \frac{\partial {\rho }}{\partial {x}} \right] = 0 \end{aligned}$$

then contains a diffusion parameter $D = C\lambda _c\rho +D_0$ that depends on $\rho$. In contrast to regular diffusion, that would disperse the axons and preclude cohesive migration, this nonlinear equation supports solutions with a relatively sharp front (as illustrated in Fig. 14), suggesting a mechanism helping leading growth cones travel together. From a biological standpoint, the main assumptions of the model have not been directly justified and are greatly idealized. However, this general mathematical approach provides an interesting foundation for a mean-field treatment of axon migration.

**Fig. 15:**
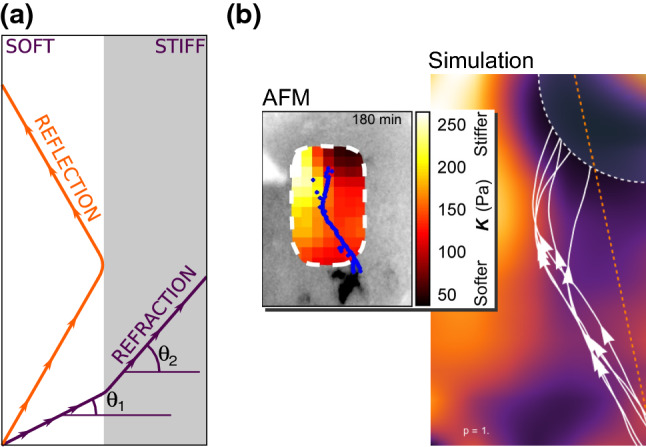
Optic-like reflection refraction of an axon bundle due to durotaxis as modeled in Oliveri et al. (2021). **a** Simulated trajectories of two bundles crossing a straight interface between a soft zone and a stiff zone. The curved portion of the trajectory represents the phase where the bundle’s cross section (not represented) has only partially crossed the interface and is therefore subject to a torque. Outside of this zone of influence, the motion is rectilinear and the incident angles $\theta _1$ and $\theta _2$ are related via a Snell-type law, which accounts for both a reflection and a refraction regime that depend on the arrival angle. **b** Deflection of the xenopus tadpole optic tract in the mid-diencephalon where we can observe a stereotypical caudal turn of the tract toward the tectum. The tract originates in the right retina and then crosses the midline in the optic chiasm, to follow the contralateral brain surface toward the left optic tectum. Inset shows the trajectory of the axon bundle (blue) and the measured stiffness of the brain surface (heat map) obtained by atomic force microscopy (AFM) on the tadpole’s brain surface (adapted from Thompson et al. 2019, under the terms of the CC BY 4.0 license). Note the contrast in stiffness between the front and rear parts of the brain surface, potentially responsible for the axon deflection (Koser et al. 2016). R.h.s. panel shows five representative simulated trajectories, with addition of noise and using a stiffness map obtained from AFM to define the stiffness field. The model, that includes only durotaxis as a guidance cue, reproduces qualitatively the trajectory of the actual optic tract, in particular the caudal turn toward the tectum that is represented by a quadrant in the top right corner

Rather than focusing on the detailed physical interactions between individual axons, it is of interest to study the motion of an entire bundle of axons viewed as a single structure. For example, a bundle was recently modeled as a tip-growing morphoelastic filament in Oliveri et al. (2021). In particular, this model focuses on durotaxis (though it is also applicable to haptotaxis) and assumes that each individual axon has a migration speed that depends on local substrate stiffness, following Chan and Odde (2008); Koch et al. (2012). Based on this idea, Koser et al postulated that gradients in tissue stiffness observed *in vivo* could induce a differential in velocity that would result in a deflection of axon tracts. In Oliveri et al. (2021), this effect is modeled by assuming that each growth cone composing the bundle’s front produces a different force depending on local substrate stiffness, which varies across the bundle’s finite width. Collectively, these forces create a torque that deflects the bundle. Surprisingly, this hypothesis leads to a behavior analogous to optic rays. For instance, at a sharp stiffness interface, a bundle is deflected according to a Snell-type law $n_1 \sin \theta _1 = n_2 \sin \theta _2$ linking the incident angles $\theta _1$ and $\theta _2$ via refractive indices $n_i$ that relate to medium compliance, as shown in Fig. 15a. Figure 15b also shows a simulation of the growing xenopus tadpole optic tract based on experimental brain stiffness data from Thompson et al. (2019). This simulation is in qualitative agreement with the observed trajectory, but does not include important factors such as chemorepulsion (Campbell et al. 2001; Piper et al. 2006; Atkinson-Leadbeater et al. 2010) or steric hindrance with other cell bodies (Koser et al. 2016). Quantitative experimental validation remains necessary to estimate the relative importance of durotaxis, among the many other cues experienced by axon tracts *in vivo*.

Note that this model represents bundles of axons growing cohesively, as seen, for example, in the xenopus optic tract (Fig. 15), but not systematically in other types of nerve. Moreover, the emergence of the exact Snell law in the model is predicated upon simple assumptions on the bundle’s mechanics (unshearable rod). In general, the bending properties of axons—*a fortiori* bundles of axons—remain poorly characterized. Nevertheless, although the model cannot capture guidance in fine details, its generality and simplicity might reveal essential properties of axon migration. In addition, the general result is potentially extendable to single axons that might follow similar optic-like behaviors at stiffness or adhesion interfaces.

### Branching and morphogenesis

**Fig. 16:**
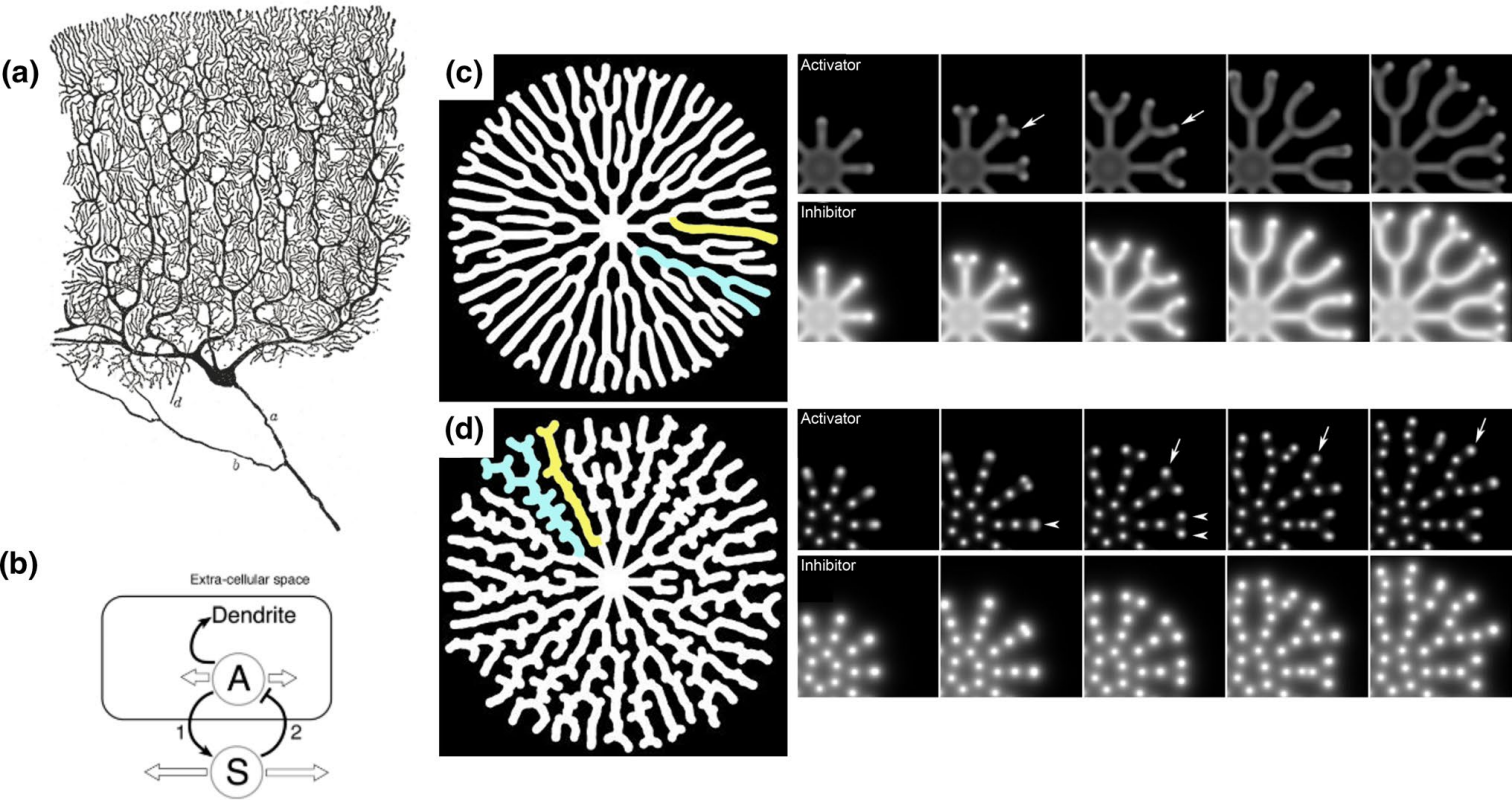
**a** Camera lucida drawing of a Purkinje cell in the cat’s cerebellar cortex by Santiago Ramón y Cajal (public domain). **b** Sugimura et al.’s activator–inhibitor model for space-filling dendrites. Intracellular activator promotes growth of dendrite and accelerates secretion of the suppressor (1). Conversely, the suppressor is secreted from the cell and diffuses in extracellular compartments. Binding of its receptor on the plasma membrane triggers signaling to inhibit synthesis of the activator (2). **c**–**d** Two simulations obtained for different values of the activator–inhibitor model, showing qualitatively different outcomes: straight branches **c** and wavy, highly branched tree **d**. For each simulation: the l.h.s. panel shows the intracellular domain in white; the r.h.s. panels show the concentration of the activator (top) and inhibitor (bottom) over time. We see that the underlying chemical patterns are very different. Images **b**–**d** adapted from Sugimura et al. (2007) under the terms of the CC BY 4.0 license

As they develop, neuronal structures, axons and dendrites, may branch and turn into more or less complex tree-like structures. Different geometries and topologies of the neuritic trees are associated with different neuron types that have been linked with different neuronal activities (Masland 2004). A striking example is provided by cerebellar Purkinje cells, shown in Fig. 16a, that exhibit remarkably large and convoluted dendritic tree structures (Kapfhammer 2004). Understanding the processes that govern neurite branching and tree formation is important to build a picture of how the neuronal circuits and nervous functions are established. In particular, the failure of these processes has been associated with developmental abnormalities and cognitive impairment as observed, for example, in Down syndrome (Haydar and Reeves 2012; Mrak and Griffin 2004).

Many generative models have been proposed to characterize and mimic the morphological diversity of neuritic trees (Ascoli 2002), using formalism such as Lindenmeyer grammars (Hamilton 1993; Ascoli 2002; Donohue et al. 2002) and other stochastic branching systems (Torben-Nielsen and De Schutter 2014; Carriquiry et al. 1991; Nowakowski et al. 1992; Uemura et al. 1995; Dityatev et al. 1995; Van Pelt et al. 1997; Van Pelt and Uylings 2005; Villacorta et al. 2007; Fujishima et al. 2012); or cellular automata (Luczak 2006; Albinet and Pelce 1996). A parsimonious example is the BESTL model which describes branching probability as a function of node depth (its *centrifugal order*) and the current number of terminal nodes in the tree (Van Pelt and Uylings 2005; Van Ooyen 2003). These approaches can reproduce observed neurons with remarkable fidelity and can be useful in applications such as classification. However, for most of them, the connection to intracellular physical mechanisms is not clearly made.

Mechanistically, neurite bifurcation occurs when the growth cone cytoskeleton splits to form two daughter branches. This event depends on several exogenous and endogenous factors (Bilimoria and Bonni 2013; Gibson and Ma 2011; Kalil et al. 2000; Kalil and Dent 2014) that have been modeled generally independently.

Several authors modeled branching as a process regulated intracellularly, and independently of the neurite’s actual spatial embedding. Graham and Van Ooyen (2004) modeled the spatially dependent concentration of tubulin subject to diffusion and active transport, where tubulin is assumed to modulate the branching probability at each time step. Mathematically, their model corresponds to an extension of the Van Veen–Van Pelt model introduced in Sect. 3.1.1, adapted to tree structure. Graham and Van Ooyen, however, impose a constant growth rate for all segments of the tree and include variations in cross-sectional sections at forking points, described by a scaling law $a_p^e = a_{c_1}^e + a_{c_2}^e$ with *e* a real exponent (Hillman 1979; Rall 1959), relating the area of the parent branch ($a_p$) to those of its two children ($a_{c_1}$ and $a_{c_2}$). Other authors have modeled the role of the MAPs (Kiddie et al. 2005; Hely et al. 2001; Graham and Van Ooyen 2004; Van Pelt et al. 2003) that are associated with increased neurite branching (Audesirk et al. 1997). Phosphorylated MAPs bind microtubules more loosely, allowing them to split. Kiddie et al. (2005); Hely et al. (2001) modeled MAP phosphorylation processes controlled by calcium in growing trees, using discrete multicompartmental approaches. For instance, Kiddie et al represented both MAP-dependent branching and tubulin assembly within the same framework.

Conversely, other authors have explored exogenous cues as the main mechanism for growth cone splitting. In this approach, it is assumed that branching results from conflicting forces applied to the growth cone (Van Veen and Van Pelt 1992; Li et al. 1995; Li and Qin 1996). Assuming that forces are applied at discrete points on the periphery of the growth cone parameterized by an angle, they define an angular distribution whose variance can be computed. In particular, a large variance signifies conflicting forces, e.g., a bimodal distribution. Branching occurs whenever this variance exceeds a given threshold, generally independently of past branching events, branch length, or current tree shape. These approaches are simple but remain phenomenological in the sense that the actual mechanical effects underlying branching are not explicitly represented and therefore are not connected to actual physical parameters such as the stiffness and viscosity of the growth cone, the adhesion with the substrate or the density of the cytoskeleton. Thus, there is an opportunity for a more detailed approach including a discussion of the actual mechanics at play in growth cone splitting.

Another remarkable phenomenon found in the morphology of neurons is the space-filling behavior of some cells, for instance, Purkinje cells (Fig. 16a). In these neurons, dendrites originating in the same cell body innervate the plane without self-overlapping (Fiala et al. 2008). This property probably facilitates efficient coverage of the space and prevents distinct branches to respond to the same input signal. Mathematically, this may be viewed as a problem of patterning with mutual avoidance, suggesting an approach based on reaction–diffusion equations. Similar to Hentschel and Fine (1996) who modeled dendritic growth by morphogen diffusion within the cytoplasm, Sugimura et al. (2007) proposed a compartmental activator–inhibitor model for dendritic self-avoidance, in which a hypothetical activator (*u*) is secreted and diffuses within the cell intracellular space. This activator then activates the production of an inhibitor (*v*), which conversely, suppresses the activator. The cell compartment is modeled by means of a density $c\left( x, y, t \right)$ obeying the local growth law

47 $$\begin{aligned} \frac{\partial {c}}{\partial {t}} = c\left( c-1 \right) \left[ a\left( u\left( x,y,t \right) \right) -c \right] , \end{aligned}$$

with $a\left( u \right) <1$, a decreasing function of *u*. A phase portrait analysis shows that growth occurs only when $a\left( u \right)$ becomes negative, i.e., when the activator *u* becomes sufficiently concentrated; otherwise $c=0$ is stable. We see that, in general, the only possible stable points are $c=0$ and $c=1$, which allows us to outline the intracellular compartment $\varOmega _c$.

Contrary to classic reaction-diffusion, the activator diffuses and reacts *only* within $\varOmega _c$. By contrast, the inhibitor diffuses in the plane $\varOmega$ allowing for extracellular interaction between distinct branches. The general mechanism is illustrated in Fig. 16b. The two quantities *u* and *v* are coupled via Turing-like equations

48 $$\begin{aligned} \frac{\partial {u}}{\partial {t}}&= D_u \nabla ^2 u + f\left( u,v \right) \quad \text {in } \varOmega _c, \end{aligned}$$

49 $$\begin{aligned} \frac{\partial {v}}{\partial {t}}&= D_v \nabla ^2 v + g\left( u,v \right) \quad \text {in } \varOmega , \end{aligned}$$

where *f* and *g* are polynomial reaction functions that satisfy basic conditions for the existence of patterns. Two simulations are shown in Fig. 16c, d. We see that the inhibitor produced by a branch diffuses in the medium and blocks the growth of neighboring branches by inhibiting the activator, thus precluding dendrite overlapping. Various parameters of the model account for qualitatively different types of dendritic trees, where the branching behavior (straight or wavy) relates to the different types of possible Turing patterns (dots or stripes). Despite its theoretical interest, this approach is relatively phenomenological and does not capture finer properties of the dendrites, such as variations in cross-sectional areas at branching loci (Hillman 1979; Tamori 1993; Rall 1959), growth inhibition between distant branches (as seen in Sect. 3.1.1), or other non-local and memory effects, such as the dependency of growth dynamics on the current topology and geometry of the tree. Furthermore, such activator–inhibitor pair, which provides a parsimonious and general mechanism for dendritic tiling, has not been identified yet *in vivo*, to our knowledge, and could likely emerge from a combination of multiple factors. Finally, contact-mediated rather than diffusion-mediated inhibition appears to be a dominant mechanism at play in space filling (Grueber and Sagasti 2010; Matthews et al. 2007; Lefebvre et al. 2012; Soba et al. 2007).

## Perspectives, challenges and opportunities

### Modeling the neurites in their environment

Our understanding of axonal growth and guidance is mostly based on controlled *in vitro* experiments where axons and dendrites evolve on a two-dimensional substrate. However, there is mounting evidence that the growth cone is a complex dynamic structure that can rapidly adapt its behavior depending on the chemical or mechanical properties of its environment. In particular, the extracellular matrix is a rich viscoelastic, three-dimensional environment that is very different from the typical substrates used in *in vitro* experiments, and where axon locomotion might be modified, as proposed by Santos et al. (2020). To understand how neurons develop, we must obtain a better picture of how neurites maneuver in complex environment, and how they interact with other cells during their development. Moreover, neurites receive multiple cues for guidance that are ultimately translated into mechanical forces for motion similar, in essence, to the problem of plant tropism (Moulton et al. 2020). Forces are probably key in orchestrating all these tropic responses. Indeed, there is mounting evidence that mechanics does not simply regulates the rate of neuronal growth, but also finely controls the nature and intensity of the response to chemical signals, suggesting that forces, mechanosensing and chemical processes are inextricably linked (see Franze 2020, and references therein).

*Challenge:* Develop multiscale and multiphysics mathematical models to understand the relationship between signal transduction, cue integration, force generation and mechanical environment in a complex three-dimensional milieu.

### Neuronal axon regeneration

**Fig. 17:**
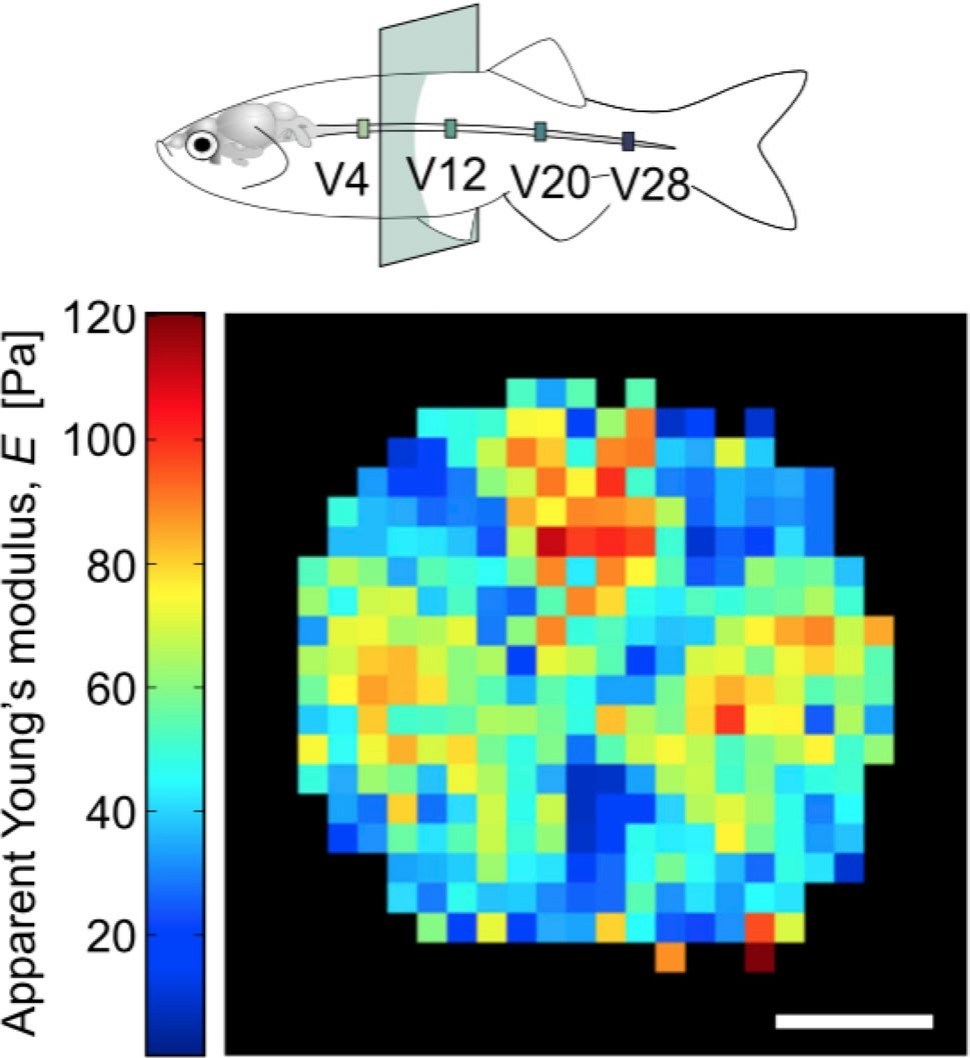
Atomic force microscopy-based indentation measurements on acute zebrafish spinal cord sections showing the spatial distribution of apparent Young’s moduli of an entire spinal cord cross section obtained from the level of the 12th vertebra. Image reproduced from Möllmert et al. (2020) with permission from the *Biophysical Society*

Axonal injuries often result in loss of function due to the disconnection of neurons. Whereas in peripheral nerves, injury may be followed by regeneration and recovery of functions, in the central nervous system of mammals, rupture often leads to permanent disabilities as these neurons fail to regenerate and reconnect to their original target in order to rebuild a proper neuronal network (Mahar and Cavalli 2018). Strangely, the situation is quite different in fish and salamanders that have the capacity for long-distance axon regeneration and functional recovery after spinal cord injury. For instance, in the zebrafish, a spinal cord injury is followed by an immediate loss of function at the injury site, but in the long run, recovery is observed, associated with a complex response involving multiple cellular pathways and morphological changes (Tsata and Wehner 2021). Clearly, this is an extremely complex process, and there is an increasing understanding that mechanics has a role to play in it. Moreover, aside from the obvious fact that forces need to be generated for motion, elongation and reconnection, the mechanical environment is also affected as the surrounding tissue has been shown to stiffen during repair (Fig. 17), as evidenced by Möllmert et al. (2020). Hence, the authors argue that mechanosensing may be important in the regeneration process and it opens the tantalizing possibility that it can be manipulated to assist spinal cord repair.

Lastly, the extraordinarily high rate at which a single cell like a neuron extend during development remains a fascinating and open problem (Smith 2009). Understanding how an axon can sustain such extreme elongation without breaking and, conversely identifying the physical processes that limit stretch induced axon elongation will be crucial in the future of regeneration therapies.

*Challenge:* Develop mathematical models that include both biochemical and mechanical responses during axonal injury and neuronal regeneration to uncover the physical mechanisms contributing to successful spinal cord repair and nerve regeneration in general.

### Growth of axons and tissues

**Fig. 18:**
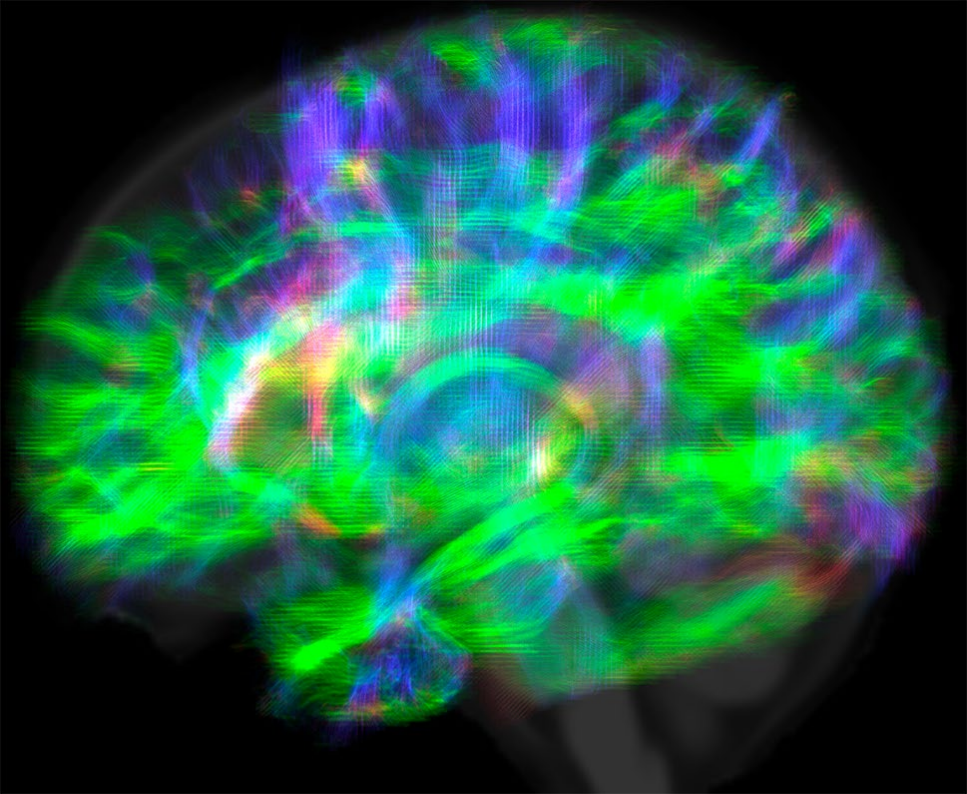
Diffusion spectrum imaging detects the movement of water molecules that flow along nerve fibers in the brain. The result is a map of the brain’s neuronal network

In the brain, the white matter (inner layer of the cortex) is mostly made up of myelinated axon tracts that connect different areas of the outer cortex, rich in neuronal bodies and part of the gray matter (Goriely et al. 2015a). Therefore, the human brain embeds an intricate and highly anisotropic arrangement of fibers, illustrated in Fig. 18. A distinct morphological trait of large mammals is a folded—or *gyrified*—brain surface. Cortical folding—*gyrification*—is a crucial and widely studied aspect of brain morphogenesis. In particular, it has received a marked interest from the biomechanics and mathematics communities (Goriely et al. 2015a, b; Garcia et al. 2018; Greiner et al. 2021; Budday et al. 2014b).

Current experimental and theoretical evidence support the hypothesis that gyrification is primarily due to a mechanical instability. The latter is triggered by the relative difference between tangential growth rates in the gray and white matter, where the fast-growing cortex comes to buckle and fold to accommodate the slow-growing subjacent tissue (Ronan and Fletcher 2015; Goriely et al. 2015b). The potential contribution of axons in controlling this instability is possible but has remained elusive despite the popularity of the idea in neurosciences (Xu et al. 2010; Bayly et al. 2014). Most mechanical models (Ben Amar and Bordner 2017; Toro and Burnod 2005; Budday et al. 2014a, 2015; Tallinen et al. 2016; Holland et al. 2015; Bayly et al. 2013; Wang et al. 2021; Budday et al. 2014b; Holland et al. 2018) are based on some version of morphoelasticity, which aims at representing the residual stresses caused by differential growth and therefore provides a natural paradigm for gyrogenesis. However, morphoelasticity is predicated upon a continuum formulation and therefore does not naturally take into account the microstructure such as axon tracts in any detail. The role of axons in gyrification has been addressed by Holland et al. (2015) who represented the preexistent pattern of nerve fibers as a field of directors defined at all points of the white matter. Based on the fact that axons grow under stretch and that they make up most of the white matter, the 3D growth tensor $\mathbf {G}$ is assumed to be anisotropically biased to occur only along the fibers, all orthogonal stretches being purely elastic. The authors propose that axon tension, albeit not being the primary cause of folding, pilots the symmetry breaking and resulting folding pattern. However, in reality, the causal link is not clear as axon paths could be partially determined by the folding pattern itself. Modern imaging techniques and detailed connectome data (Fig. 18), in tandem with modeling, might prove valuable in studying the precise timing of these correlated events.

*Challenge:* Develop mathematical methods to explore the trajectories of migrating axons in the entire brain cortex, and the possible links between gyrification and fiber network geometry.

## Conclusion

We have reviewed key mathematical models aimed at answering questions regarding neurite growth, pathfinding and patterning. Many of the fundamental mechanisms underlying these processes are only partially understood or remain elusive, and this is where mathematical modeling can play a key role. It allows for systematic testing of hypotheses and, with the emergence of quantitative biophysical models, can be used to identify fundamental mechanisms. In this regard, we want to emphasize the importance of mechanics in this process. Growth, morphogenesis, guidance all rely on the generation and sensing of forces that need to be appropriately taken into account. While simple zero-dimensional models are suitable to build intuition, we now understand that, from a mechanical point of view, these structures are not rigid but active morphoelastic materials that combine properties of fluids and solids together with active force generation, internal remodeling, and growth, requiring advanced mathematical models.

Many other models focus on transport processes or biochemical patterning. These different approaches offer snapshots of properties that must be combined in order to obtain a global picture. Indeed neurites are complex, dynamic and pluriform structures. Thus, future models must integrate the many rules that govern neurite protrusion, extension, sensing, collapse, stalling, steering, exploration, branching, fasciculation and defasciculation. The standard experimental approach is to isolate different effects to understand a particular mechanism. By contrast, mathematical modeling, combined with numerical simulations and systematic validation, has the potential to integrate these effects in order to understand the emergence of complex morphologies and behaviors.

*“Upstairs, my little noseminers! Go! Flee before me! Onward and upward! Go pump some neurons. Expand your craniums.”* Robin Williams, *Mrs. Doubtfire*, 1993.

## References

[CR1] Abuwarda H, Pathak MM (2020) Mechanobiology of neural development. Curr Opin Cell Biol 66:104–111. 10.1016/j.ceb.2020.05.012 32687993 10.1016/j.ceb.2020.05.012 PMC7578076

[CR2] Aeschlimann M (2000) Biophysical models of axonal pathfinding. PhD thesis, University of Lausanne

[CR3] Aeschlimann M, Tettoni L (2001) Biophysical model of axonal pathfinding. Neurocomputing 38–40:87–92. 10.1016/S0925-2312(01)00539-2 10.1016/S0925-2312(01)00539-2

[CR253] Ahmadzadeh H, Smith DH, Shenoy VB (2015) Mechanical effects of dynamic binding between Tau proteins on microtubules during axonal injury. Biophys J 109(11):2328–2337. 10.1016/j.bpj.2015.09.010 26636944 10.1016/j.bpj.2015.09.010 PMC4675823

[CR4] Ahmed WW, Saif TA (2014) Active transport of vesicles in neurons is modulated by mechanical tension. Sci Rep 4(1):1–7. 10.1038/srep04481 10.1038/srep04481 PMC3967286 24670781

[CR5] Albinet G, Pelce P (1996) Computer simulation of neurite outgrowth. Europhys Lett 33(7):569–574. 10.1209/epl/i1996-00380-5 10.1209/epl/i1996-00380-5

[CR6] Aletti G, Causin P, Naldi G (2008) A model for axon guidance: sensing, transduction and movement. AIP Conf Proc 1028:129–146. 10.1063/1.2965082 10.1063/1.2965082

[CR7] Ali O, Mirabet V, Godin C, Traas J (2014) Physical models of plant development. Annu Rev Cell Dev Biol 30(1):59–78. 10.1146/annurev-cellbio-101512-122410 25000996 10.1146/annurev-cellbio-101512-122410

[CR8] Ambrosi D, Ben Amar M, Cyron CJ, De Simone A, Goriely A, Humphrey JD, Kuhl E (2019) Growth and remodelling of living tissues: perspectives, challenges and opportunities. J R Soc Interface 16(157):20190233. 10.1098/rsif.2019.0233 31431183 10.1098/rsif.2019.0233 PMC6731508

[CR9] Anthonisen M, Grütter P (2019) Growth and elasticity of mechanically-created neurites. arXiv preprint arXiv:1912.05735 10.1016/j.jmbbm.2019.06.015 31229904

[CR10] Ascoli GA (2002) Neuroanatomical algorithms for dendritic modelling. Netw Comput Neural Syst 13(3):247–260. 10.1088/0954-898X_13_3_301 10.1088/0954-898X_13_3_301 12222813

[CR11] Athamneh AI, Suter DM (2015) Quantifying mechanical force in axonal growth and guidance. Front Cell Neurosci 9:359. 10.3389/fncel.2015.00359 26441530 10.3389/fncel.2015.00359 PMC4584967

[CR12] Athamneh AI, He Y, Lamoureux P, Fix L, Suter DM, Miller KE (2017) Neurite elongation is highly correlated with bulk forward translocation of microtubules. Sci Rep 7(1):1–13. 10.1038/s41598-017-07402-6 28779177 10.1038/s41598-017-07402-6 PMC5544698

[CR13] Atkinson-Leadbeater K, Bertolesi GE, Hehr CL, Webber CA, Cechmanek PB, McFarlane S (2010) Dynamic expression of axon guidance cues required for optic tract development is controlled by fibroblast growth factor signaling. J Neurosci 30(2):685–693. 10.1523/JNEUROSCI.4165-09.2010 20071533 10.1523/JNEUROSCI.4165-09.2010 PMC6633001

[CR14] Audesirk G, Cabell L, Kern M (1997) Modulation of neurite branching by protein phosphorylation in cultured rat hippocampal neurons. Dev Brain Res 102(2):247–260. 10.1016/S0165-3806(97)00100-4 9352107 10.1016/S0165-3806(97)00100-4

[CR15] Azevedo FA, Carvalho LR, Grinberg LT, Farfel JM, Ferretti RE, Leite RE, Filho WJ, Lent R, Herculano-Houzel S (2009) Equal numbers of neuronal and nonneuronal cells make the human brain an isometrically scaled-up primate brain. J Comp Neurol 513(5):532–541. 10.1002/cne.21974 19226510 10.1002/cne.21974

[CR16] Bamburg J, Bray D, Chapman K (1986) Assembly of microtubules at the tip of growing axons. Nature 321(6072):788–790 2872595 10.1038/321788a0

[CR17] Bangasser BL, Odde DJ (2013) Master equation-based analysis of a motor-clutch model for cell traction force. Cell Mol Bioeng 6(4):449–459. 10.1007/s12195-013-0296-5 24465279 10.1007/s12195-013-0296-5 PMC3896613

[CR19] Basso JMV, Yurchenko I, Wiens MR, Staii C (2019) Neuron dynamics on directional surfaces. Soft Matter 15(48):9931–9941. 10.1039/C9SM01769K 31764921 10.1039/C9SM01769K

[CR20] Bayly PV, Okamoto RJ, Xu G, Shi Y, Taber LA (2013) A cortical folding model incorporating stress-dependent growth explains gyral wavelengths and stress patterns in the developing brain. Phys Biol 10(1):016005. 10.1088/1478-3975/10/1/016005 23357794 10.1088/1478-3975/10/1/016005 PMC3616769

[CR21] Bayly PV, Taber LA, Kroenke CD (2014) Mechanical forces in cerebral cortical folding: a review of measurements and models. J Mech Behav Biomed Mater 29:568–581. 10.1016/j.jmbbm.2013.02.018 23566768 10.1016/j.jmbbm.2013.02.018 PMC3842388

[CR22] Ben Amar M, Bordner A (2017) Mimicking cortex convolutions through the wrinkling of growing soft bilayers. J Elast 129(1–2):213–238. 10.1007/s10659-017-9622-9 10.1007/s10659-017-9622-9

[CR23] Berg HC, Purcell EM (1977) Physics of chemoreception. Biophys J 20(2):193–219. 10.1016/S0006-3495(77)85544-6 911982 10.1016/S0006-3495(77)85544-6 PMC1473391

[CR24] Bernal R, Pullarkat PA, Melo F (2007) Mechanical properties of axons. Phys Rev E 99(1):018301. 10.1103/PhysRevLett.99.018301 10.1103/PhysRevLett.99.018301 17678192

[CR25] Betz T, Koch D, Lim D, Käs JA (2009) Stochastic actin polymerization and steady retrograde flow determine growth cone advancement. Biophys J 96(12):5130–5138. 10.1016/j.bpj.2009.03.045 19527673 10.1016/j.bpj.2009.03.045 PMC2712033

[CR26] Bilimoria PM, Bonni A (2013) Molecular control of axon branching. Neuroscientist 19(1):16–24. 10.1177/1073858411426201 22179123 10.1177/1073858411426201 PMC3490022

[CR27] Black MM, Slaughter T, Fischer I (1994) Microtubule-associated protein 1b (map1b) is concentrated in the distal region of growing axons. J Neurosci 14(2):857–870 8301365 10.1523/JNEUROSCI.14-02-00857 PMC6576811

[CR28] Borisyuk R, Cooke T, Roberts A (2008) Stochasticity and functionality of neural systems: mathematical modelling of axon growth in the spinal cord of tadpole. Biosystems 93(1–2):101–114. 10.1016/j.biosystems.2008.03.012 18547713 10.1016/j.biosystems.2008.03.012

[CR29] Boudon F, Chopard J, Ali O, Gilles B, Hamant O, Boudaoud A, Traas J, Godin C (2015) A computational framework for 3D mechanical modeling of plant morphogenesis with cellular resolution. PLoS Comput Biol. 10.1371/journal.pcbi.1003950 10.1371/journal.pcbi.1003950 PMC4288716 25569615

[CR30] Bozorg B, Krupinski P, Jönsson H (2016) A continuous growth model for plant tissue. Phys Biol 13(6):65002. 10.1088/1478-3975/13/6/065002 10.1088/1478-3975/13/6/065002 27845935

[CR31] Bray D (1984) Axonal growth in response to experimentally applied mechanical tension. Dev Biol 102(2):379–389. 10.1016/0012-1606(84)90202-1 6706005 10.1016/0012-1606(84)90202-1

[CR32] Bressloff PC, Newby JM (2013) Stochastic models of intracellular transport. Rev Mod Phys 85(1):135–196. 10.1103/RevModPhys.85.135 10.1103/RevModPhys.85.135

[CR33] Budday S, Raybaud C, Kuhl E (2014) A mechanical model predicts morphological abnormalities in the developing human brain. Sci Rep 4(1):1–7. 10.1038/srep05644 10.1038/srep05644 PMC4090617 25008163

[CR34] Budday S, Steinmann P, Kuhl E (2014) The role of mechanics during brain development. J Mech Phys Solids 72:75–92. 10.1016/j.jmps.2014.07.010 25202162 10.1016/j.jmps.2014.07.010 PMC4156279

[CR35] Budday S, Steinmann P, Goriely A, Kuhl E (2015) Size and curvature regulate pattern selection in the mammalian brain. Extreme Mech Lett 4:193–198. 10.1016/j.eml.2015.07.004 10.1016/j.eml.2015.07.004

[CR36] Buettner HM, Pittman RN, Ivins JK (1994) A model of neurite extension across regions of nonpermissive substrate: simulations based on experimental measurement of growth cone motility and filopodial dynamics. Dev Biol 163(2):407–422. 10.1006/dbio.1994.1158 8200479 10.1006/dbio.1994.1158

[CR37] Buxbaum R, Heidemann S (1988) A thermodynamic model for force integration and microtubule assembly during axonal elongation. J Theor Biol 134(3):379–390. 10.1016/S0022-5193(88)80068-7 3254435 10.1016/S0022-5193(88)80068-7

[CR38] Buxbaum RE, Heidemann SR (1992) An absolute rate theory model for tension control of axonal elongation. J Theor Biol. 10.1016/S0022-5193(05)80626-5 10.1016/S0022-5193(05)80626-5 1619959

[CR39] Campbell DS, Regan AG, Lopez JS, Tannahill D, Harris WA, Holt CE (2001) Semaphorin 3a elicits stage-dependent collapse, turning, and branching in xenopus retinal growth cones. J Neurosci 21(21):8538–8547. 10.1523/JNEUROSCI.21-21-08538.2001 11606642 10.1523/JNEUROSCI.21-21-08538.2001 PMC6762807

[CR40] Carriquiry AL, Ireland WP, Kliemann W, Uemura E (1991) Statistical evaluation of dendritic growth models. Bull Math Biol 53(4):579–589. 10.1007/BF02458630 1933030 10.1007/BF02458630

[CR41] Caviston JP, Holzbaur EL (2006) Microtubule motors at the intersection of trafficking and transport. Trends Cell Biol 16(10):530–537. 10.1016/j.tcb.2006.08.002 16938456 10.1016/j.tcb.2006.08.002

[CR42] Chan CE, Odde DJ (2008) Traction dynamics of filopodia on compliant substrates. Science 322(5908):1687–1691. 10.1126/science.1163595 19074349 10.1126/science.1163595

[CR43] Chaudhuri D, Borowski P, Zapotocky M (2011) Model of fasciculation and sorting in mixed populations of axons. Phys Rev E 84(2):021908. 10.1103/PhysRevE.84.021908 10.1103/PhysRevE.84.021908 21929021

[CR44] Chilton JK (2006) Molecular mechanisms of axon guidance. Dev Biol 292(1):13–24. 10.1016/j.ydbio.2005.12.048 16476423 10.1016/j.ydbio.2005.12.048

[CR45] Coles CH, Bradke F (2015) Coordinating neuronal actin-microtubule dynamics. Curr Biol 25(15):R677–R691. 10.1016/j.cub.2015.06.020 26241148 10.1016/j.cub.2015.06.020

[CR46] Davis O, Merrison-Hort R, Soffe SR, Borisyuk R (2017) Studying the role of axon fasciculation during development in a computational model of the Xenopus tadpole spinal cord. Sci Rep 7(1):1–16. 10.1038/s41598-017-13804-3 29051550 10.1038/s41598-017-13804-3 PMC5648846

[CR47] De Gennes PG (2007) Collective neuronal growth and self organization of axons. Proc Natl Acad Sci 104(12):4904–4906. 10.1073/pnas.0609871104 17360378 10.1073/pnas.0609871104 PMC1829237

[CR48] De Rooij R, Kuhl E (2018) Microtubule polymerization and cross-link dynamics explain axonal stiffness and damage. Biophys J 114(1):201–212. 10.1016/j.bpj.2017.11.010 29320687 10.1016/j.bpj.2017.11.010 PMC5773766

[CR49] De Rooij R, Kuhl E (2018) Physical biology of axonal damage. Front Cell Neurosci 12:144. 10.3389/fncel.2018.00144 29928193 10.3389/fncel.2018.00144 PMC5997835

[CR50] De Rooij R, Miller KE, Kuhl E (2017) Modeling molecular mechanisms in the axon. Comput Mech 59(3):523–537. 10.1007/s00466-016-1359-y 28603326 10.1007/s00466-016-1359-y PMC5464742

[CR51] De Rooij R, Kuhl E, Miller KE (2018) Modeling the axon as an active partner with the growth cone in axonal elongation. Biophys J 115(9):1783–1795. 10.1016/j.bpj.2018.08.047 30309611 10.1016/j.bpj.2018.08.047 PMC6224630

[CR52] Dennerll TJ, Lamoureux P, Buxbaum RE, Heidemann SR (1989) The cytomechanics of axonal elongation and retraction. J Cell Biol 109 (6 I):3073–3083. 10.1083/jcb.109.6.3073 2592415 10.1083/jcb.109.6.3073 PMC2115908

[CR53] Dent EW, Gertler FB (2003) Cytoskeletal dynamics and transport in growth cone motility and axon guidance. Neuron 40(2):209–227. 10.1016/S0896-6273(03)00633-0 14556705 10.1016/S0896-6273(03)00633-0

[CR54] Dickson BJ (2002) Molecular mechanisms of axon guidance. Science 298(5600):1959–1964. 10.1126/science.1072165 12471249 10.1126/science.1072165

[CR55] Diehl S, Henningsson E, Heyden A, Perna S (2014) A one-dimensional moving-boundary model for tubulin-driven axonal growth. J Theor Biol 358:194–207. 10.1016/j.jtbi.2014.06.019 24956328 10.1016/j.jtbi.2014.06.019

[CR56] Diehl S, Henningsson E, Heyden A (2016) Efficient simulations of tubulin-driven axonal growth. J Comput Neurosci 41(1):45–63. 10.1007/s10827-016-0604-x 27121476 10.1007/s10827-016-0604-x

[CR57] Dityatev AE, Chmykhova NM, Studer L, Karamian OA, Kozhanov VM, Clamann HP (1995) Comparison of the topology and growth rules of motoneuronal dendrites. J Comp Neurol 363(3):505–516. 10.1002/cne.903630312 8847414 10.1002/cne.903630312

[CR58] Donohue DE, Scorcioni R, Ascoli GA (2002) Generation and description of neuronal morphology using L-Neuron. Computational neuroanatomy. Springer, Heidelberg, pp 49–69

[CR59] Espina JA, Marchant CL, Barriga EH (2021) Durotaxis: the mechanical control of directed cell migration. FEBS J. 10.1111/febs.15862 10.1111/febs.15862 PMC9292038 33811732

[CR60] Fanti Z, De-Miguel FF, Martinez-Perez ME (2008) A method for semiautomatic tracing and morphological measuring of neurite outgrowth from dic sequences. In: 2008 30th annual international conference of the IEEE engineering in medicine and biology society, pp 1196–1199. 10.1109/IEMBS.2008.4649377 10.1109/IEMBS.2008.4649377 19162880

[CR61] Fiala JC, Spacek J, Harris KM (2008) Dendrite structure. In: Stuart G, Spruston N, Häusser M (eds) Dendrites, 2nd edn, Oxford University Press, chap 1, pp 1–41. 10.1093/acprof:oso/9780198566564.003.0001

[CR62] Fivaz M, Bandara S, Inoue T, Meyer T (2008) Robust neuronal symmetry breaking by Ras-triggered local positive feedback. Curr Biol 18(1):44–50. 10.1016/j.cub.2007.11.051 18158244 10.1016/j.cub.2007.11.051

[CR63] Forbes EM, Thompson AW, Yuan J, Goodhill GJ (2012) Calcium and cAMP levels interact to determine attraction versus repulsion in axon guidance. Neuron 74(3):490–503. 10.1016/j.neuron.2012.02.035 22578501 10.1016/j.neuron.2012.02.035

[CR64] Franze K (2020) Integrating chemistry and mechanics: the forces driving axon growth. Annu Rev Cell Dev Biol. 10.1146/annurev-cellbio-100818-125157 10.1146/annurev-cellbio-100818-125157 32603614

[CR65] Franze K, Guck J (2010) The biophysics of neuronal growth. Rep on Prog Phys. 10.1088/0034-4885/73/9/094601 10.1088/0034-4885/73/9/094601

[CR66] Franze K, Gerdelmann J, Weick M, Betz T, Pawlizak S, Lakadamyali M, Bayer J, Rillich K, Gögler M, Lu YB et al (2009) Neurite branch retraction is caused by a threshold-dependent mechanical impact. Biophys J 97(7):1883–1890. 10.1016/j.bpj.2009.07.033 19804718 10.1016/j.bpj.2009.07.033 PMC2756359

[CR67] Franze K, Reichenbach A, Käs J (2009) Biomechanics of the CNS. In: Kamkim A, Kiseleva I (eds) Mechanosensitivity of the nervous system mechanosensitivity in cells and tissues. Springer, Heidelberg, pp 173–213

[CR68] Franze K, Janmey PA, Guck J (2013) Mechanics in neuronal development and repair. Annu Rev Biomed Eng 15(1):227–251. 10.1146/annurev-bioeng-071811-150045 23642242 10.1146/annurev-bioeng-071811-150045

[CR69] Fujishima K, Horie R, Mochizuki A, Kengaku M (2012) Principles of branch dynamics governing shape characteristics of cerebellar purkinje cell dendrites. Development 139(18):3442–3455. 10.1242/dev.081315 22912417 10.1242/dev.081315 PMC3491647

[CR70] Futerman AH, Banker GA (1996) The economics of neurite outgrowth–the addition of new membrane to growing axons. Trends Neurosci 19(4):144–149. 10.1016/s0166-2236(96)80025-7 8658598 10.1016/s0166-2236(96)80025-7

[CR71] Garcia K, Kroenke C, Bayly P (2018) Mechanics of cortical folding: stress, growth and stability. Philoso Trans R Soc B: Biol Sci 373(1759):20170321. 10.1098/rstb.2017.0321 10.1098/rstb.2017.0321 PMC6158197 30249772

[CR72] García-Grajales JA, Peña JM, McHugh S, Jérusalem A (2012) A model of the spatially dependent mechanical properties of the axon during its growth. CMES - Comput Model Eng Sci 87(5):411–432. 10.3970/cmes.2012.087.411 10.3970/cmes.2012.087.411

[CR73] García-Grajales JA, Jérusalem A, Goriely A (2017) Continuum mechanical modeling of axonal growth. Comput Methods Appl Mech Eng 314:147–163. 10.1016/j.cma.2016.07.032 10.1016/j.cma.2016.07.032

[CR74] Gay DA, Sisodia SS, Cleveland DW (1989) Autoregulatory control of -tubulin mRNA stability is linked to translation elongation. Proc Natl Acad Sci USA 86(15):5763–5767. 10.1073/pnas.86.15.5763 2762294 10.1073/pnas.86.15.5763 PMC297710

[CR75] Gibson DA, Ma L (2011) Developmental regulation of axon branching in the vertebrate nervous system. Development 138(2):183–195. 10.1242/dev.046441 21177340 10.1242/dev.046441 PMC3005597

[CR76] Gierer A, Meinhardt H (1972) A theory of biological pattern formation. Kybernetik 39:30–39. 10.1007/BF00289234 10.1007/BF00289234 4663624

[CR77] Giniūnaité R, Baker RE, Kulesa PM, Maini PK (2020) Modelling collective cell migration: neural crest as a model paradigm. J Math Biol 80(1):481–504. 10.1007/s00285-019-01436-2 31587096 10.1007/s00285-019-01436-2 PMC7012984

[CR78] Gokoffski KK, Jia X, Shvarts D, Xia G, Zhao M (2019) Physiologic electrical fields direct retinal ganglion cell axon growth *in vitro*. Invest Ophthalmol Vis Sci 60(10):3659–3668. 10.1167/iovs.18-25118 31469406 10.1167/iovs.18-25118 PMC6716951

[CR79] Goldberg DJ, Burmeister DW (1986) Stages in axon formation: observations of growth of Aplysia axons in culture using video-enhanced contrast-differential interference contrast microscopy. J Cell Biol 103(5):1921–1931. 10.1083/jcb.103.5.1921 3782290 10.1083/jcb.103.5.1921 PMC2114395

[CR80] Goodhill GJ (1997) Diffusion in axon guidance. Eur J Neurosci 9(7):1414–1421. 10.1111/j.1460-9568.1997.tb01496.x 9240399 10.1111/j.1460-9568.1997.tb01496.x

[CR81] Goodhill GJ (1998) Mathematical guidance for axons. Trends Neurosci 21(6):226–231. 10.1016/S0166-2236(97)01203-4 9641531 10.1016/S0166-2236(97)01203-4

[CR82] Goodhill GJ (2018) Theoretical models of neural development. iScience 8:183–199. 10.1016/j.isci.2018.09.017 30321813 10.1016/j.isci.2018.09.017 PMC6197653

[CR83] Goodhill GJ, Baier H (1998) Axon guidance: stretching gradients to the limit. Neural Comput 10(3):521–527. 10.1162/089976698300017638 9527831 10.1162/089976698300017638

[CR84] Goodhill GJ, Urbach JS (1999) Theoretical analysis of gradient detection by growth cones. J Neurobiol 41(2):230–241 10.1002/(SICI)1097-4695(19991105)41:2<230::AID-NEU6>3.0.CO;2-9 10512980 10.1002/(SICI)1097-4695(19991105)41:2<230::AID-NEU6>3.0.CO;2-9

[CR85] Goodhill GJ, Urbach JS (2003) Axon guidance and gradient detection by growth cones. In: Modeling Neural (ed) Van Ooyen A. The MIT Press, Development, Cambridge, pp 95–109

[CR86] Goodhill GJ, Gu M, Urbach JS (2004) Predicting axonal response to molecular gradients with a computational model of filopodial dynamics. Neural Comput 16(11):2221–2243. 10.1162/0899766041941934 15476599 10.1162/0899766041941934

[CR87] Goriely A (2017) The mathematics and mechanics of biological growth, Interdisciplinary applied mathematics, vol 45, 1st edn. Springer-Verlag, New York

[CR88] Goriely A (2018) Five ways to model active processes in elastic solids: active forces, active stresses, active strains, active fibers, and active metrics. Mech Res Commun 93:75–79. 10.1016/j.mechrescom.2017.09.003 10.1016/j.mechrescom.2017.09.003

[CR89] Goriely A, Robertson-Tessi M, Tabor M, Vandiver R (2008) Elastic growth models. In: Mondaini RP, Pardalos PM (eds) Mathematical modelling of biosystems, applied optimization, vol 102. Springer, Berlin Heidelberg, pp 1–44

[CR90] Goriely A, Budday S, Kuhl E (2015) Neuromechanics: from neurons to brain. Advances in applied mechanics, vol 48. Academic Press Inc., Cambridge, pp 79–139

[CR91] Goriely A, Geers MG, Holzapfel GA, Jayamohan J, Jérusalem A, Sivaloganathan S, Squier W, Van Dommelen JA, Waters S, Kuhl E (2015) Mechanics of the brain: perspectives, challenges, and opportunities. Biomech Model Mechanobiol 14(5):931–965. 10.1007/s10237-015-0662-4 25716305 10.1007/s10237-015-0662-4 PMC4562999

[CR92] Graham BP, Van Ooyen A (2001) Compartmental models of growing neurites. Neurocomputing 38–40:31–36. 10.1016/S0925-2312(01)00463-5 10.1016/S0925-2312(01)00463-5

[CR93] Graham BP, Van Ooyen A (2004) Transport limited effects in a model of dendritic branching. J Theor Biol 230:421–432. 10.1016/j.jtbi.2004.06.007 15321709 10.1016/j.jtbi.2004.06.007

[CR94] Graham BP, Van Ooyen A (2006) Mathematical modelling and numerical simulation of the morphological development of neurons. BMC Neurosci. 10.1186/1471-2202-7-S1-S9 10.1186/1471-2202-7-S1-S9 PMC1679805 17118163

[CR95] Graham BP, Lauchlan K, Mclean DR (2006) Dynamics of outgrowth in a continuum model of neurite elongation. J Comput Neurosci 20(1):43–60. 10.1007/s10827-006-5330-3 16649067 10.1007/s10827-006-5330-3

[CR96] Greiner A, Kaessmair S, Budday S (2021) Physical aspects of cortical folding. Soft Matter 17(5):1210–1222. 10.1039/D0SM02209H 33480902 10.1039/D0SM02209H

[CR97] Grueber WB, Sagasti A (2010) Self-avoidance and tiling: mechanisms of dendrite and axon spacing. Cold Spring Harb Perspect Biol 2(9):a001750. 10.1101/cshperspect.a001750 20573716 10.1101/cshperspect.a001750 PMC2926746

[CR98] Hamant O (2017) Mechano-devo. Mech Dev 145:2–9. 10.1016/j.mod.2017.02.004 28315388 10.1016/j.mod.2017.02.004

[CR99] Hamid S, Hayek R (2008) Role of electrical stimulation for rehabilitation and regeneration after spinal cord injury: an overview. Eur Spine J 17(9):1256–1269. 10.1007/s00586-008-0729-3 18677518 10.1007/s00586-008-0729-3 PMC2527422

[CR100] Hamilton P (1993) A language to describe the growth of neurites. Biol Cybern 68(6):559–565. 10.1007/BF00200816 8324064 10.1007/BF00200816

[CR101] Haydar TF, Reeves RH (2012) Trisomy 21 and early brain development. Trends Neurosci 35(2):81–91. 10.1016/j.tins.2011.11.001 22169531 10.1016/j.tins.2011.11.001 PMC3273608

[CR102] Heidemann SR, Buxbaum RE (1990) Tension as a regulator and integrator of axonal growth. Cell Motil Cytoskelet 17(1):6–10. 10.1002/cm.970170103 10.1002/cm.970170103 2225090

[CR103] Heidemann SR, Lamoureux P, Buxbaum RE (1990) Growth cone behavior and production of traction force. J Cell Biol 111(5):1949–1957 2229183 10.1083/jcb.111.5.1949 PMC2116337

[CR104] Heidemann SR, Lamoureux P, Buxbaum RE (1997) Cytomechanics of axonal development. Cell Biochem Biophys 27(3):135–155. 10.1007/BF02738107 10.1007/BF02738107 9279454

[CR105] Hely TA, Van Ooyen A, Willshaw DJ (1998) A simulation of growth cone filopodia dynamics based on turing morphogenesis patterns. In: Holcombe M, Paton R (eds) Information processing in cells and tissues. Springer, Heidelberg, pp 69–73

[CR106] Hely TA, Graham B, Van Ooyen A (2001) A computational model of dendrite elongation and branching based on MAP2 phosphorylation. J Theor Biol 210(3):375–384. 10.1006/jtbi.2001.2314 11397138 10.1006/jtbi.2001.2314

[CR107] Hentschel HGE, Fine A (1996) Diffusion-regulated control of cellular dendritic morphogenesis. Proc R Soc B Biol Sci 263(1366):1–8. 10.1098/rspb.1996.0001 10.1098/rspb.1996.0001 8587892

[CR108] Hentschel HGE, Van Ooyen A (1999) Models of axon guidance and bundling during development. Proc R Soc B Biol Sci 266(1434):2231–2238. 10.1098/rspb.1999.0913 10.1098/rspb.1999.0913 PMC1690341 10649638

[CR109] Hentschel HGE, Van Ooyen A (2000) Dynamic mechanisms for bundling and guidance during neural network formation. Phys A 288(1–4):369–379. 10.1016/S0378-4371(00)00434-9 10.1016/S0378-4371(00)00434-9

[CR110] Hillman D (1979) Neuronal shape parameters and substructures as a basis of neuronal form. In: Schmitt FO, Worden FG (eds) The neurosciences, fourth study program. MIT Press, Cambridge, MA, pp 477–498

[CR111] Hjorth JJ, Van Pelt J, Mansvelder HD, Van Ooyen A (2014) Competitive dynamics during resource-driven neurite outgrowth. PLoS One 9(2):86741. 10.1371/journal.pone.0086741 10.1371/journal.pone.0086741 PMC3911915 24498280

[CR112] Hoffman PN, Griffin JW, Gold BG, Price DL (1985) Slowing of neurofilament transport and the radial growth of developing nerve fibers. J Neurosci 5(11):2920–2929. 10.1523/jneurosci.05-11-02920.1985 2414416 10.1523/jneurosci.05-11-02920.1985 PMC6565160

[CR113] Holland M, Budday S, Goriely A, Kuhl E (2018) Symmetry breaking in wrinkling patterns: gyri are universally thicker than sulci. Phys Rev Lett 121(22):228002. 10.1103/PhysRevLett.121.228002 30547630 10.1103/PhysRevLett.121.228002

[CR114] Holland MA, Miller KE, Kuhl E (2015) Emerging brain morphologies from axonal elongation. Ann Biomed Eng 43(7):1640–1653. 10.1007/s10439-015-1312-9 25824370 10.1007/s10439-015-1312-9 PMC4497873

[CR115] Holzapfel G (2000) Nonlinear solid mechanics: a continuum approach for engineering science. Wiley, Hoboken

[CR116] Inagaki N, Toriyama M, Sakumura Y (2011) Systems biology of symmetry breaking during neuronal polarity formation. Dev Neurobiol 71(6):584–593. 10.1002/dneu.20837 21557507 10.1002/dneu.20837

[CR117] Jakobs M, Franze K, Zemel A (2015) Force generation by molecular-motor-powered microtubule bundles; implications for neuronal polarization and growth. Front Cell Neurosci 9:441. 10.3389/fncel.2015.00441 26617489 10.3389/fncel.2015.00441 PMC4639704

[CR118] Jakobs MA, Franze K, Zemel A (2020) Mechanical regulation of neurite polarization and growth: a computational study. Biophys J 118(8):1914–1920. 10.1016/j.bpj.2020.02.031 32229314 10.1016/j.bpj.2020.02.031 PMC7175593

[CR119] Janulevicius A, van Pelt J, van Ooyen A (2006) Compartment volume influences microtubule dynamic instability: a model study. Biophys J 90(3):788–798. 10.1529/biophysj.105.059410 16410484 10.1529/biophysj.105.059410 PMC1367104

[CR120] Jülicher F, Kruse K, Prost J, Joanny JF (2007) Active behavior of the cytoskeleton. Phys Rep 449(1–3):3–28. 10.1016/j.physrep.2007.02.018 10.1016/j.physrep.2007.02.018

[CR121] Kalil K, Dent EW (2014) Branch management: mechanisms of axon branching in the developing vertebrate CNS. Nat Rev Neurosci 15(1):7–18. 10.1038/nrn3650 24356070 10.1038/nrn3650 PMC4063290

[CR122] Kalil K, Szebenyi G, Dent EW (2000) Common mechanisms underlying growth cone guidance and axon branching. J Neurobiol 44(2):145–158 10934318 10.1002/1097-4695(200008)44:2<145::AID-NEU5>3.0.CO;2-X

[CR123] Kapfhammer JP (2004) Cellular and molecular control of dendritic growth and development of cerebellar Purkinje cells. Prog Histochem Cytochem 39(3):131–182. 10.1016/j.proghi.2004.07.002 15580762 10.1016/j.proghi.2004.07.002

[CR124] Katz MJ (1985) How straight do axons grow? J Neurosci 5(3):589–595. 10.1523/jneurosci.05-03-00589.1985 3973686 10.1523/jneurosci.05-03-00589.1985 PMC6565018

[CR125] Katz MJ, George EB, Gilbert LJ (1984) Axonal elongation as a stochastic walk. Cell Motil 4(5):351–370. 10.1002/cm.970040505 6509522 10.1002/cm.970040505

[CR126] Kevenaar JT, Hoogenraad CC (2015) The axonal cytoskeleton: from organization to function. Front Mol Neurosci 8:44. 10.3389/fnmol.044 26321907 10.3389/fnmol.044 PMC4536388

[CR127] Kiddie G, McLean D, Van Ooyen A, Graham B (2005) Biologically plausible models of neurite outgrowth. Development, dynamics and pathiology of neuronal networks: from molecules to functional circuits, progress in brain research. Elsevier, Amesterdam, pp 67–80 10.1016/S0079-6123(04)47006-X 15581698

[CR128] Kiryushko D, Berezin V, Bock E (2004) Regulators of neurite outgrowth: role of cell adhesion molecules. Ann N Y Acad Sci 1014(1):140–154. 10.1196/annals.1294.015 15153429 10.1196/annals.1294.015

[CR129] Koch D, Rosoff WJ, Jiang J, Geller HM, Urbach JS (2012) Strength in the periphery: growth cone biomechanics and substrate rigidity response in peripheral and central nervous system neurons. Biophys J 102(3):452–460. 10.1016/j.bpj.2011.12.025 22325267 10.1016/j.bpj.2011.12.025 PMC3274825

[CR130] Koser DE, Thompson AJ, Foster SK, Dwivedy A, Pillai EK, Sheridan GK, Svoboda H, Viana M, da Fontoura Costa L, Guck J, Holt CE, Franze K (2016) Mechanosensing is critical for axon growth in the developing brain. Nat Neurosci 19(12):1592. 10.1038/nn.4394 27643431 10.1038/nn.4394 PMC5531257

[CR131] Krottje JK, Van Ooyen A (2007) A mathematical framework for modeling axon guidance. Bull Math Biol 69(1):3–31. 10.1007/s11538-006-9142-4 17061055 10.1007/s11538-006-9142-4 PMC2806218

[CR132] Lamoureux P, Buxbaum RE, Heidemann SR (1989) Direct evidence that growth cones pull. Nature 340(6229):159–162. 10.1038/340159a0 2739738 10.1038/340159a0

[CR133] Lamoureux P, Heidemann SR, Martzke NR, Miller KE (2010) Growth and elongation within and along the axon. Dev Neurobiol 70(3):135–149. 10.1002/dneu.20764 19950193 10.1002/dneu.20764

[CR134] Lefebvre JL, Kostadinov D, Chen WV, Maniatis T, Sanes JR (2012) Protocadherins mediate dendritic self-avoidance in the mammalian nervous system. Nature 488(7412):517–521. 10.1038/nature11305 22842903 10.1038/nature11305 PMC3427422

[CR135] Li GH, Qin CD (1996) A model for neurite growth and neuronal morphogenesis. Math Biosci 132(1):97–110. 10.1016/0025-5564(95)00052-6 8924723 10.1016/0025-5564(95)00052-6

[CR136] Li GH, Qin CD, Wang ZS (1992) Neurite branching pattern formation: modeling and computer simulation. J Theor Biol 157(4):463–486. 10.1016/S0022-5193(05)80664-2 1460876 10.1016/S0022-5193(05)80664-2

[CR137] Li GH, Wang LW et al (1995) Computer model of growth cone behavior and neuronal morphogenesis. J Theor Biol 174(4):381–389. 10.1006/jtbi.1995.0106 10.1006/jtbi.1995.0106

[CR138] Lim SS, Edson KJ, Letourneau PC, Borisy GG (1990) A test of microtubule translocation during neurite elongation. J Cell Biol 111(1):123–130 2195037 10.1083/jcb.111.1.123 PMC2116169

[CR139] Lin J, Li X, Yin J, Qian J (2020) Effect of cyclic stretch on neuron reorientation and axon outgrowth. Front Bioeng Biotechnol 8:1429. 10.3389/fbioe.2020.597867 10.3389/fbioe.2020.597867 PMC7793818 33425865

[CR140] Lockhart JA (1965) An analysis of irreversible plant cell elongation. J Theor Biol 8(2):264–275. 10.1016/0022-5193(65)90077-9 5876240 10.1016/0022-5193(65)90077-9

[CR141] Loverde JR, Ozoka VC, Aquino R, Lin L, Pfister BJ (2011) Live imaging of axon stretch growth in embryonic and adult neurons. J Neurotrauma 28(11):2389–2403. 10.1089/neu.2010.1598 21663384 10.1089/neu.2010.1598

[CR142] Luczak A (2006) Spatial embedding of neuronal trees modeled by diffusive growth. J Neurosci Methods 157(1):132–141. 10.1016/j.jneumeth.2006.03.024 16690135 10.1016/j.jneumeth.2006.03.024

[CR143] Maccioni RB, Cambiazo V (1995) Role of microtubule-associated proteins in the control of microtubule assembly. Physiol Rev 75(4):835–864. 10.1152/physrev.1995.75.4.835 7480164 10.1152/physrev.1995.75.4.835

[CR144] Mahar M, Cavalli V (2018) Intrinsic mechanisms of neuronal axon regeneration. Nat Rev Neurosci 19(6):323–337. 10.1038/s41583-018-0001-8 29666508 10.1038/s41583-018-0001-8 PMC5987780

[CR145] Maskery S, Shinbrot T (2005) Deterministic and stochastic elements of axonal guidance. Annu Rev Biomed Eng 7:187–221. 10.1146/annurev.bioeng.7.060804.100446 16004570 10.1146/annurev.bioeng.7.060804.100446

[CR146] Maskery SM, Buettner HM, Shinbrot T (2004) Growth cone pathfinding: a competition between deterministic and stochastic events. BMC Neurosci 5(1):22. 10.1186/1471-2202-5-22 15242518 10.1186/1471-2202-5-22 PMC499546

[CR147] Masland RH (2004) Neuronal cell types. Curr Biol 14(13):R497–R500. 10.1016/j.cub.2004.06.035 15242626 10.1016/j.cub.2004.06.035

[CR148] Matthews BJ, Kim ME, Flanagan JJ, Hattori D, Clemens JC, Zipursky SL, Grueber WB (2007) Dendrite self-avoidance is controlled by Dscam. Cell 129(3):593–604. 10.1016/j.cell.2007.04.013 17482551 10.1016/j.cell.2007.04.013

[CR149] McCormick LE, Gupton SL (2020) Mechanistic advances in axon pathfinding. Curr Opin Cell Biol 63:11–19. 10.1016/j.ceb.2019.12.003 31927278 10.1016/j.ceb.2019.12.003 PMC7247931

[CR150] McLean DR, Graham BP (2004) Mathematical formulation and analysis of a continuum model for tubulin-driven neurite elongation. Proc R Soc A Math Phys Eng Sci 460(2048):2437–2456. 10.1098/rspa.2004.1288 10.1098/rspa.2004.1288

[CR151] McLean DR, Graham BP (2006) Stability in a mathematical model of neurite elongation. Math Med Biol J IMA 23(2):101–117. 10.1093/imammb/dql010 10.1093/imammb/dql010 16672287

[CR152] McLean DR, Van Ooyen A, Graham BP (2004) Continuum model for tubulin-driven neurite elongation. Neurocomputing 58–60:511–516. 10.1016/j.neucom.2004.01.088 10.1016/j.neucom.2004.01.088

[CR153] Meinhardt H, Gierer A (2000) Pattern formation by local self-activation and lateral inhibition. BioEssays 22(8):753–760 10918306 10.1002/1521-1878(200008)22:8<lt;753::AID-BIES9>3.0.CO;2-Z

[CR154] Miller KE, Heidemann SR (2008) What is slow axonal transport? Exp Cell Res 314(10):1981–1990. 10.1016/j.yexcr.2008.03.004 18410924 10.1016/j.yexcr.2008.03.004

[CR155] Miller KE, Joshi HC (1996) Tubulin transport in neurons. J Cell Biol 133(6):1355–1366. 10.1083/jcb.133.6.1355 8682870 10.1083/jcb.133.6.1355 PMC2120892

[CR156] Miller KE, Samuels DC (1997) The axon as a metabolic compartment: protein degradation, transport, and maximum length of an axon. J Theor Biol 186(3):373–379. 10.1006/jtbi.1996.0355 9219672 10.1006/jtbi.1996.0355

[CR157] Miller KE, Sheetz MP (2006) Direct evidence for coherent low velocity axonal transport of mitochondria. J Cell Biol 173(3):373–381. 10.1083/jcb.200510097 16682527 10.1083/jcb.200510097 PMC2063838

[CR158] Miller KE, Suter DM (2018) An integrated cytoskeletal model of neurite outgrowth. Front Cell Neurosci. 10.3389/fncel.2018.00447 10.3389/fncel.2018.00447 PMC6275320 30534055

[CR159] Möllmert S, Kharlamova MA, Hoche T, Taubenberger AV, Abuhattum S, Kuscha V, Kurth T, Brand M, Guck J (2020) Zebrafish spinal cord repair is accompanied by transient tissue stiffening. Biophys J 118(2):448–463. 10.1016/j.bpj.2019.10.044 31870536 10.1016/j.bpj.2019.10.044 PMC6976874

[CR251] Montanino A, Kleiven S (2018) Utilizing a structural mechanics approach to assess the primary effects of injury loads onto the axon and its components. Front Neurol. 10.3389/fneur.2018.00643 10.3389/fneur.2018.00643 PMC6087765 30127763

[CR160] Mortimer D, Fothergill T, Pujic Z, Richards LJ, Goodhill GJ (2008) Growth cone chemotaxis. Trends Neurosci 31(2):90–98. 10.1016/j.tins.2007.11.008 18201774 10.1016/j.tins.2007.11.008

[CR161] Mortimer D, Pujic Z, Vaughan T, Thompson AW, Feldner J, Vetter I, Goodhill GJ (2010) Axon guidance by growth-rate modulation. Proc Natl Acad Sci 107(11):5202–5207. 10.1073/pnas.0909254107 20194766 10.1073/pnas.0909254107 PMC2841880

[CR162] Moulton DE, Lessinnes T, Goriely A (2013) Morphoelastic rods. Part I: a single growing elastic rod. J Mech Phys Solids 61(2):398–427. 10.1016/j.jmps.2012.09.017 10.1016/j.jmps.2012.09.017

[CR163] Moulton DE, Oliveri H, Goriely A (2020) Multiscale integration of environmental stimuli in plant tropism produces complex behaviors. Proc Natl Acad Sci 117(51):32226–32237. 10.1073/PNAS.2016025117 33273121 10.1073/PNAS.2016025117 PMC7768784

[CR164] Mrak RE, Griffin WST (2004) Trisomy 21 and the brain. J Neuropathol Exp Neurol 63(7):679–685. 10.1093/jnen/63.7.679 15290893 10.1093/jnen/63.7.679 PMC3833615

[CR165] Murray JD (1993) Mathematical Biology, 2nd edn. Springer-Verlag, Berlin

[CR166] Mutalik SP, Ghose A (2020) Axonal cytomechanics in neuronal development. J Biosci 45(1):1–17. 10.1007/s12038-020-00029-2 32385223 10.1007/s12038-020-00029-2

[CR167] Nowakowski RS, Hayes NL, Egger MD (1992) Competitive interactions during dendritic growth: a simple stochastic growth algorithm. Brain Res 576(1):152–156. 10.1016/0006-8993(92)90622-g 1381258 10.1016/0006-8993(92)90622-g

[CR168] O’Donnell M, Chance RK, Bashaw GJ (2009) Axon growth and guidance: receptor regulation and signal transduction. Annu Rev Neurosci 32(1):383–412. 10.1146/annurev.neuro.051508.135614 19400716 10.1146/annurev.neuro.051508.135614 PMC4765433

[CR169] Okabe S, Hirokawa N (1990) Turnover of fluorescently labelled tubulin and actin in the axon. Nature 343(6257):479–482. 10.1038/343479a0 1689016 10.1038/343479a0

[CR170] Oliveri H, Franze K, Goriely A (2021) Theory for durotactic axon guidance. Phys Rev Lett 126(11):118101. 10.1103/PhysRevLett.126.118101 33798338 10.1103/PhysRevLett.126.118101

[CR171] O’Toole M, Miller KE (2011) The role of stretching in slow axonal transport. Biophys J 100(2):351–360. 10.1016/j.bpj.2010.12.3695 21244831 10.1016/j.bpj.2010.12.3695 PMC3021655

[CR172] O’Toole M, Lamoureux P, Miller KE (2008) A physical model of axonal elongation: force, viscosity, and adhesions govern the mode of outgrowth. Biophys J 94(7):2610–2620. 10.1529/biophysj.107.117424 18178646 10.1529/biophysj.107.117424 PMC2267140

[CR173] O’Toole M, Latham R, Baqri RM, Miller KE (2008) Modeling mitochondrial dynamics during *in vivo* axonal elongation. J Theor Biol 255(4):369–377. 10.1016/j.jtbi.2008.09.009 18845167 10.1016/j.jtbi.2008.09.009

[CR174] O’Toole M, Lamoureux P, Miller KE (2015) Measurement of subcellular force generation in neurons. Biophys J 108(5):1027–1037. 10.1016/j.bpj.2015.01.021 25762315 10.1016/j.bpj.2015.01.021 PMC4375613

[CR175] Painter KJ (2019) Mathematical models for chemotaxis and their applications in self-organisation phenomena. J Theor Biol 481:162–182. 10.1016/j.jtbi.2018.06.019 29944856 10.1016/j.jtbi.2018.06.019

[CR176] Pearson YE, Castronovo E, Lindsley TA, Drew DA (2011) Mathematical modeling of axonal formation part I: geometry. Bull Math Biol 73(12):2837–2864. 10.1007/s11538-011-9648-2 21390561 10.1007/s11538-011-9648-2

[CR177] Peter SJ, Mofrad MR (2012) Computational modeling of axonal microtubule bundles under tension. Biophys J 102(4):749–757. 10.1016/J.BPJ.2011.11.4024 22385845 10.1016/J.BPJ.2011.11.4024 PMC3283805

[CR178] Pfister BJ, Iwata A, Meaney DF, Smith DH (2004) Extreme stretch growth of integrated axons. J Neurosci 24(36):7978–7983. 10.1523/JNEUROSCI.1974-04.2004 15356212 10.1523/JNEUROSCI.1974-04.2004 PMC6729931

[CR179] Pillay S, Byrne HM, Maini PK (2017) Modeling angiogenesis: a discrete to continuum description. Phys Rev E 95(1):12410. 10.1103/PhysRevE.95.012410 10.1103/PhysRevE.95.012410 28208423

[CR180] Piper M, Anderson R, Dwivedy A, Weinl C, van Horck F, Leung KM, Cogill E, Holt C (2006) Signaling mechanisms underlying slit2-induced collapse of xenopus retinal growth cones. Neuron 49(2):215–228. 10.1016/j.neuron.2005.12.008 16423696 10.1016/j.neuron.2005.12.008 PMC3689199

[CR181] Plachez C, Richards LJ (2005) Mechanisms of axon guidance in the developing nervous system. Curr Top Dev Biol 69:267–346. 10.1016/S0070-2153(05)69010-2 16243603 10.1016/S0070-2153(05)69010-2

[CR182] Purohit PK (2015) Tension dependent growth and retraction of neurites. Proced IUTAM 12:185–192. 10.1016/j.piutam.2014.12.020 10.1016/j.piutam.2014.12.020

[CR183] Purohit PK, Smith DH (2016) A model for stretch growth of neurons. J Biomech 49(16):3934–3942. 10.1016/j.jbiomech.2016.11.045 27890538 10.1016/j.jbiomech.2016.11.045 PMC8710257

[CR252] Rajagopalan J, Tofangchi A, Saif MTA (2010) Drosophila neurons actively regulate axonal tension in vivo. Biophys J 99(10):3208–3215. 10.1016/j.bpj.2010.09.029 21081068 10.1016/j.bpj.2010.09.029 PMC2980728

[CR184] Rakic P (1972) Mode of cell migration to the superficial layers of fetal monkey neocortex. J Comp Neurol 145(1):61–83. 10.1002/cne.901450105 4624784 10.1002/cne.901450105

[CR185] Rall W (1959) Branching dendritic trees and motoneuron membrane resistivity. Exp Neurol 1(5):491–527. 10.1016/0014-4886(59)90046-9 14435979 10.1016/0014-4886(59)90046-9

[CR186] Recho P, Jérusalem A, Goriely A (2016) Growth, collapse, and stalling in a mechanical model for neurite motility. Phys Rev E. 10.1103/PhysRevE.93.032410 10.1103/PhysRevE.93.032410 27078393

[CR187] Ren Y, Suter DM (2016) Increase in growth cone size correlates with decrease in neurite growth rate. Neural Plast 2016:20–22. 10.1155/2016/3497901 10.1155/2016/3497901 PMC4870373 27274874

[CR188] Riccobelli D (2021) Active elasticity drives the formation of periodic beading in damaged axons. Phys Rev E 104(024):417. 10.1103/PhysRevE.104.024417 10.1103/PhysRevE.104.024417 34525524

[CR189] Roberts A, Conte D, Hull M, Merrison-Hort R, al Azad AK, Buhl E, Borisyuk R, Soffe SR (2014) Can simple rules control development of a pioneer vertebrate neuronal network generating behavior? J Neurosci 34(2):608–621. 10.1523/JNEUROSCI.3248-13.2014 24403159 10.1523/JNEUROSCI.3248-13.2014 PMC3870938

[CR190] Roccasalvo IM, Micera S, Sergi PN (2015) A hybrid computational model to predict chemotactic guidance of growth cones. Sci Rep 5(1):1–17. 10.1038/srep11340 10.1038/srep11340 PMC4471899 26086936

[CR191] Rodriguez EK, Hoger A, McCulloch AD (1994) Stress-dependent finite growth in soft elastic tissues. J Biomech 27(4):455–467. 10.1016/0021-9290(94)90021-3 8188726 10.1016/0021-9290(94)90021-3

[CR192] Ronan L, Fletcher PC (2015) From genes to folds: a review of cortical gyrification theory. Brain Struct Funct 220(5):2475–2483. 10.1007/s00429-014-0961-z 25511709 10.1007/s00429-014-0961-z PMC4549381

[CR193] Roy S (2014) Seeing the unseen: the hidden world of slow axonal transport. Neuroscientist 20(1):71–81. 10.1177/1073858413498306 23912032 10.1177/1073858413498306 PMC3902140

[CR194] Roy S (2020) Finding order in slow axonal transport. Curr Opin Neurobiol 63:87–94. 10.1016/j.conb.2020.03.015 32361600 10.1016/j.conb.2020.03.015 PMC7483916

[CR195] Sabry J, O’Connor TP, Kirschner MW (1995) Axonal transport of tubulin in tit pioneer neurons *in situ*. Neuron 14(6):1247–1256. 10.1016/0896-6273(95)90271-6 7541635 10.1016/0896-6273(95)90271-6

[CR196] Samuels DC, Hentschel H, Fine A (1996) The origin of neuronal polarization: a model of axon formation. Philos Trans R Soc Lond B Biol Sci 351(1344):1147–1156. 10.1098/rstb.1996.0099 8899865 10.1098/rstb.1996.0099

[CR197] Santos TE, Schaffran B, Broguière N, Meyn L, Zenobi-Wong M, Bradke F (2020) Axon growth of CNS neurons in three dimensions is amoeboid and independent of adhesions. Cell Rep 32(3):107907. 10.1016/j.celrep.2020.107907 32698008 10.1016/j.celrep.2020.107907

[CR198] Segev R, Ben-Jacob E (2000) Generic modeling of chemotactic based self-wiring of neural networks. Neural Netw 13(2):185–199. 10.1016/S0893-6080(99)00084-2 10935760 10.1016/S0893-6080(99)00084-2

[CR199] Segev R, Ben-Jacob E (2001) Chemical waves and internal energy during cooperative self-wiring of neural nets. Neurocomputing 38–40:875–879. 10.1016/S0925-2312(01)00369-1 10.1016/S0925-2312(01)00369-1

[CR200] Seo J, Youn W, Choi JY, Cho H, Choi H, Lanara C, Stratakis E, Choi IS (2020) Neuro-taxis: neuronal movement in gradients of chemical and physical environments. Dev Neurobiol 80(9–10):361–377. 10.1002/dneu.22749 32304173 10.1002/dneu.22749

[CR201] Shapiro S, Borgens R, Pascuzzi R, Roos K, Groff M, Purvines S, Rodgers RB, Hagy S, Nelson P (2005) Oscillating field stimulation for complete spinal cord injury in humans: a phase 1 trial. J Neurosurg Spine 2(1):3–10. 10.3171/spi.2005.2.1.0003 15658119 10.3171/spi.2005.2.1.0003

[CR202] Sherratt JA, Murray JD (1990) Models of epidermal wound healing. Proc R Soc Lond B 241(1300):29–36. 10.1098/rspb.1990.0061 10.1098/rspb.1990.0061 1978332

[CR203] Siechen S, Yang S, Chiba A, Saif T (2009) Mechanical tension contributes to clustering of neurotransmitter vesicles at presynaptic terminals. Proc Natl Acad Sci 106(31):12611–12616. 10.1073/pnas.0901867106 19620718 10.1073/pnas.0901867106 PMC2713391

[CR204] Simpson HD, Mortimer D, Goodhill GJ (2009) Theoretical models of neural circuit development. Development of neural circuitry,current topics in developmental biology. Academic Press, Cambridge, pp 1–51 10.1016/S0070-2153(09)01201-0 19427515

[CR205] Smeal RM, Rabbitt R, Biran R, Tresco PA (2005) Substrate curvature influences the direction of nerve outgrowth. Ann Biomed Eng 33(3):376–382. 10.1007/s10439-005-1740-z 15868728 10.1007/s10439-005-1740-z

[CR206] Smith DA, Simmons RM (2001) Models of motor-assisted transport of intracellular particles. Biophys J 80(1):45–68. 10.1016/S0006-3495(01)75994-2 11159382 10.1016/S0006-3495(01)75994-2 PMC1301213

[CR207] Smith DH (2009) Stretch growth of integrated axon tracts: extremes and exploitations. Prog Neurobiol 89(3):231–239. 10.1016/j.pneurobio.2009.07.006 19664679 10.1016/j.pneurobio.2009.07.006 PMC3019093

[CR208] Soba P, Zhu S, Emoto K, Younger S, Yang SJ, Yu HH, Lee T, Jan LY, Jan YN (2007) Drosophila sensory neurons require Dscam for dendritic self-avoidance and proper dendritic field organization. Neuron 54(3):403–416. 10.1016/j.neuron.2007.03.029 17481394 10.1016/j.neuron.2007.03.029 PMC1963441

[CR209] Striedter GF (2016) Neurobiology: a functional approach. Oxford University Press, Oxford

[CR210] Sugimura K, Shimono K, Uemura T, Mochizuki A (2007) Self-organizing mechanism for development of space-filling neuronal dendrites. PLoS Comput Biol 3(11):2143–2154. 10.1371/journal.pcbi.0030212 10.1371/journal.pcbi.0030212 PMC2077899 18020700

[CR211] Sundararaghavan HG, Masand SN, Shreiber DI (2011) Microfluidic generation of haptotactic gradients through 3D collagen gels for enhanced neurite growth. J Neurotrauma 28(11):2377–2387. 10.1089/neu.2010.1606 21473683 10.1089/neu.2010.1606 PMC3218382

[CR212] Suter DM, Miller KE (2011) The emerging role of forces in axonal elongation. Prog Neurobiol 94(2):91–101. 10.1016/j.pneurobio.2011.04.002 21527310 10.1016/j.pneurobio.2011.04.002 PMC3115633

[CR213] Sutherland DJ, Pujic Z, Goodhill GJ (2014) Calcium signaling in axon guidance. Trends Neurosci 37(8):424–432. 10.1016/j.tins.2014.05.008 24969461 10.1016/j.tins.2014.05.008

[CR214] Takano T, Xu C, Funahashi Y, Namba T, Kaibuchi K (2015) Neuronal polarization. Development 142(12):2088–2093. 10.1242/dev.114454 26081570 10.1242/dev.114454

[CR215] Takano T, Wu M, Nakamuta S, Naoki H, Ishizawa N, Namba T, Watanabe T, Xu C, Hamaguchi T, Yura Y, Amano M, Hahn KM, Kaibuchi K (2017) Discovery of long-range inhibitory signaling to ensure single axon formation. Nat Commun 8(1):1–17. 10.1038/s41467-017-00044-2 28652571 10.1038/s41467-017-00044-2 PMC5484694

[CR216] Takeda S, Funakoshi T, Hirokawa N (1995) Tubulin dynamics in neuronal axons of living zebrafish embryos. Neuron 14(6):1257–1264. 10.1016/0896-6273(95)90272-4 7541636 10.1016/0896-6273(95)90272-4

[CR217] Tallinen T, Chung JY, Rousseau F, Girard N, Lefèvre J, Mahadevan L (2016) On the growth and form of cortical convolutions. Nat Phys 12(6):588–593. 10.1038/nphys3632 10.1038/nphys3632

[CR218] Tamori Y (1993) Theory of dendritic morphology. Phys Rev E 48(4):3124–3129. 10.1103/PhysRevE.48.3124 10.1103/PhysRevE.48.3124 9960951

[CR219] Thompson AJ, Pillai EK, Dimov IB, Foster SK, Holt CE, Franze K (2019) Rapid changes in tissue mechanics regulate cell behaviour in the developing embryonic brain. eLife 8:e39356. 10.7554/eLife.39356 30642430 10.7554/eLife.39356 PMC6333438

[CR220] Thompson DM, Buettner HM (2006) Neurite outgrowth is directed by schwann cell alignment in the absence of other guidance cues. Ann Biomed Eng 34(1):161. 10.1007/s10439-005-9013-4 16453203 10.1007/s10439-005-9013-4

[CR221] Torben-Nielsen B, De Schutter E (2014) Context-aware modeling of neuronal morphologies. Front Neuroanat. 10.3389/fnana.2014.00092 10.3389/fnana.2014.00092 PMC4155795 25249944

[CR222] Toriyama M, Sakumura Y, Shimada T, Ishii S, Inagaki N (2010) A diffusion-based neurite length-sensing mechanism involved in neuronal symmetry breaking. Mol Syst Biol 6(394):1–16. 10.1038/msb.2010.51 10.1038/msb.2010.51 PMC2925530 20664640

[CR223] Toro R, Burnod Y (2005) A morphogenetic model for the development of cortical convolutions. Cereb Cortex 15(12):1900–1913. 10.1093/cercor/bhi068 15758198 10.1093/cercor/bhi068

[CR224] Tsata V, Wehner D (2021) Know how to regrow–axon regeneration in the zebrafish spinal cord. Cells 10(6):1404. 10.3390/cells10061404 34204045 10.3390/cells10061404 PMC8228677

[CR225] Turing AM (1952) The chemical basis of morphogenesis. Philos Trans R Soc (part B) 52(641):37–72 10.1007/BF02459572

[CR226] Uemura E, Carriquiry A, Kliemann W, Goodwin J (1995) Mathematical modeling of dendritic growth *in vitro*. Brain Res 671(2):187–194. 10.1016/0006-8993(94)01310-E 7743207 10.1016/0006-8993(94)01310-E

[CR227] Van Ooyen A (2003) Modeling neural development. The MIT Press, Cambridge

[CR228] Van Ooyen A (2011) Using theoretical models to analyse neural development. Nat Rev Neurosci 12(6):311–326. 10.1038/nrn3031 21587288 10.1038/nrn3031

[CR229] Van Ooyen A, Graham B, Ramakers GJ (2001) Competition for tubulin between growing neurites during development. Neurocomputing 38–40:73–78. 10.1016/S0925-2312(01)00487-8 10.1016/S0925-2312(01)00487-8

[CR230] Van Pelt J, Uylings HB (2005) Natural variability in the geometry of dendritic branching patterns. Modeling in the neurosciences. CRC Press, Boca Raton, pp 107–134

[CR231] Van Pelt J, Dityatev AE, Uylings HB (1997) Natural variability in the number of dendritic segments: model-based inferences about branching during neurite outgrowth. J Comp Neurol 387(3):325–340 9335418 10.1002/(SICI)1096-9861(19971027)387:3<325::AID-CNE1>3.0.CO;2-2

[CR232] Van Pelt J, Graham BP, Uylings HB (2003) Formation of dendritic branching patterns. In: Van Ooyen A (ed) Modeling neural development. The MIT Press, Cambridge, pp 75–94

[CR233] Van Vactor D (1998) Adhesion and signaling in axonal fasciculation. Curr Opin Neurobiol 8(1):80–86. 10.1016/s0959-4388(98)80011-1 9568395 10.1016/s0959-4388(98)80011-1

[CR234] Van Veen M, Van Pelt J (1992) A model for outgrowth of branching neurites. J Theor Biol 159(1):1–23. 10.1016/S0022-5193(05)80764-7 10.1016/S0022-5193(05)80764-7

[CR235] Van Veen M, Van Pelt J (1994) Neuritic growth rate described by modeling microtubule dynamics. Bull Math Biol 56(2):249–273. 10.1016/s0092-8240(05)80258-7 8186754 10.1016/s0092-8240(05)80258-7

[CR236] Vicsek T, Zafeiris A (2012) Collective motion. Phys Rep 517(3–4):71–140. 10.1016/j.physrep.2012.03.004 10.1016/j.physrep.2012.03.004

[CR237] Villacorta JA, Castro J, Negredo P, Avendaño C (2007) Mathematical foundations of the dendritic growth models. J Math Biol 55(5):817–859. 10.1007/s00285-007-0113-7 17646989 10.1007/s00285-007-0113-7

[CR238] Wang FS, Liu CW, Diefenbach TJ, Jay DG (2003) Modeling the role of myosin 1c in neuronal growth cone turning. Biophys J 85(5):3319–3328. 10.1016/S0006-3495(03)74751-1 14581233 10.1016/S0006-3495(03)74751-1 PMC1303609

[CR239] Wang LM, Kuhl E (2019) Viscoelasticity of the axon limits stretch-mediated growth. Comput Mech 65:587–595. 10.1007/s00466-019-01784-2 10.1007/s00466-019-01784-2

[CR240] Wang S, Demirci N, Holland MA (2021) Numerical investigation of biomechanically coupled growth in cortical folding. Biomech Model Mechanobiol 20(2):555–567. 10.1007/s10237-020-01400-w 33151429 10.1007/s10237-020-01400-w

[CR241] Watson DF, Hoffman PN, Fittro KP, Griffin JW (1989) Neurofilament and tubulin transport slows along the course of mature motor axons. Brain Res 477(1–2):225–232. 10.1016/0006-8993(89)91410-8 2467723 10.1016/0006-8993(89)91410-8

[CR242] Xu J, Rosoff WJ, Urbach JS, Goodhill GJ (2005) Adaptation is not required to explain the long-term response of axons to molecular gradients. Development 132(20):4545–4552. 10.1242/dev.02029 16176951 10.1242/dev.02029

[CR243] Xu G, Knutsen AK, Dikranian K, Kroenke CD, Bayly PV, Taber LA (2010) Axons pull on the brain, but tension does not drive cortical folding. J Biomech Eng 132(7):071013. 10.1115/1.4001683 20590291 10.1115/1.4001683 PMC3170872

[CR244] Yao L, Li Y (2016) The role of direct current electric field-guided stem cell migration in neural regeneration. Stem Cell Rev Rep 12(3):365–375. 10.1007/s12015-016-9654-8 27108005 10.1007/s12015-016-9654-8

[CR245] Yurchenko I, Vensi Basso JM, Syrotenko VS, Staii C (2019) Anomalous diffusion for neuronal growth on surfaces with controlled geometries. PLoS One 14(5):e0216181. 10.1371/journal.pone.0216181 31059532 10.1371/journal.pone.0216181 PMC6502317

[CR246] Yurchenko I, Farwell M, Brady DD, Staii C (2021) Neuronal growth and formation of neuron networks on directional surfaces. Biomimetics. 10.3390/biomimetics6020041 10.3390/biomimetics6020041 PMC8293217 34208649

[CR247] Zadeh KS, Shah SB (2010) Mathematical modeling and parameter estimation of axonal cargo transport. J Comput Neurosci 28(3):495–507. 10.1007/s10827-010-0232-9 20407814 10.1007/s10827-010-0232-9

[CR248] Zhao F, Du F, Oliveri H, Zhou L, Ali O, Chen W, Feng S, Wang Q, Lü S, Long M, Schneider R, Sampathkumar A, Godin C, Traas J, Jiao Y (2020) Microtubule-mediated wall anisotropy contributes to leaf blade flattening. Curr Biol 30(20):3972–3985.10.1016/j.cub.2020.07.076 32916107 10.1016/j.cub.2020.07.076 PMC7575199

[CR249] Zheng J, Lamoureux P, Santiago V, Dennerll T, Buxbaum RE, Heidemann SR (1991) Tensile regulation of axonal elongation and initiation. J Neurosci 11(4):1117–1125. 10.1523/JNEUROSCI.11-04-01117.1991 2010807 10.1523/JNEUROSCI.11-04-01117.1991 PMC6575379

[CR250] Zubler F, Douglas R (2009) A framework for modeling the growth and development of neurons and networks. Front Comput Neurosci 3:25. 10.3389/neuro.10.025.2009 19949465 10.3389/neuro.10.025.2009 PMC2784082

