# Correction to: Mathematical models of neuronal growth

**DOI:** 10.1007/s10237-024-01831-9

**Published:** 2024-03-29

**Authors:** Hadrien Oliveri, Alain Goriely

**Affiliations:** 1 Max-Planck-Institut für molekulare Zellbiologie und Genetik, 01307 Dresden, Germany; 2 Center for Systems Biology Dresden, 01307 Dresden, Germany; 3 Fakultät Mathematik, Technische Universität Dresden, 01062 Dresden, Germany; 4 Mathematical Institute, University of Oxford, Oxford, OX2 6GG UK

## Abstract

**Correction to: Biomechanics and Modeling in Mechanobiology (2022) 21:89–118** https://doi.org/10.1007/s10237-021-01539-0

We report some minor errors in the article titled “Mathematical models of neuronal growth” by Hadrien Oliveri and Alain Goriely, published in *Biomechanics and Modeling in Mechanobiology* on 7 July 2022 (Oliveri and Goriely 2022):

1. Equation (4) should be replaced by:
  $$\begin{aligned} \frac{\textrm{d}c_1}{\textrm{d}t} = \frac{J}{V} + k^- - k^+ c_1. \end{aligned}$$
  This adjustment ensures that $k^-$ has the dimension of a concentration per unit time, while $k^+$ has the dimension of an inverse time. This is consistent with Equation (2), where we introduced the parameter *e* with dimension of time per concentration.
2. On page 92, a proper nondimensionalisation can be achieved by introducing the respective time, length, and concentration units *A*/*D*, *SAe*/*VD*, and *SA*/*VD* (correcting the initially chosen length unit). This revised nondimensionalisation introduces the dimensionless parameters $\alpha = A D / Se$, $\beta = V k^-/S$, and $\gamma = Ak^+/D$. It is important to note that this correction does not affect the nondimensionalised equations or the results depicted in the figures for given values of $\alpha$, $\beta$, and $\gamma$.
3. The following statement “*It is well known that there is no physical (positive) solution to the diffusion equation in one or two dimensions*” should be corrected to read “There is no physical (positive) *steady* solution to the diffusion equation with a constant point source in one and two dimensions”.
